# Surveillance for Violent Deaths — National Violent Death Reporting System, 39 States, the District of Columbia, and Puerto Rico, 2018

**DOI:** 10.15585/mmwr.ss7103a1

**Published:** 2022-01-28

**Authors:** Kameron J. Sheats, Rebecca F. Wilson, Bridget H. Lyons, Shane P.D. Jack, Carter J. Betz, Katherine A. Fowler

**Affiliations:** 1 Division of Violence Prevention, National Center for Injury Prevention and Control, CDC

## Abstract

**Problem/Condition:**

In 2018, approximately 68,000 persons died of violence-related injuries in the United States. This report summarizes data from CDC’s National Violent Death Reporting System (NVDRS) on violent deaths that occurred in 39 states the District of Columbia, and Puerto Rico in 2018. Results are reported by sex, age group, race and ethnicity, method of injury, type of location where the injury occurred, circumstances of injury, and other selected characteristics.

**Period Covered:**

2018.

**Description of System:**

NVDRS collects data regarding violent deaths obtained from death certificates, coroner and medical examiner reports, and law enforcement reports. This report includes data collected for violent deaths that occurred in 2018. Data were collected from 36 states with statewide data (Alabama, Alaska, Arizona, Colorado, Connecticut, Delaware, Georgia, Indiana, Iowa, Kansas, Kentucky, Louisiana, Maine, Maryland, Massachusetts, Michigan, Minnesota, Missouri, Nebraska, Nevada, New Hampshire, New Jersey, New Mexico, New York, North Carolina, Ohio, Oklahoma, Oregon, Rhode Island, South Carolina, Utah, Vermont, Virginia, Washington, West Virginia, and Wisconsin), three states with data from counties representing a subset of their population (21 California counties, 28 Illinois counties, and 39 Pennsylvania counties), the District of Columbia, and Puerto Rico. NVDRS collates information for each death and links deaths that are related (e.g., multiple homicides, homicide followed by suicide, or multiple suicides) into a single incident.

**Results:**

For 2018, NVDRS collected information on 52,773 fatal incidents involving 54,170 deaths that occurred in 39 states and the District of Columbia. In addition, information was collected on 880 fatal incidents involving 975 deaths in Puerto Rico. Data for Puerto Rico were analyzed separately. Of the 54,170 deaths, the majority (64.1%) were suicides, followed by homicides (24.8%), deaths of undetermined intent (9.0%), legal intervention deaths (1.4%) (i.e., deaths caused by law enforcement and other persons with legal authority to use deadly force acting in the line of duty, excluding legal executions), and unintentional firearm deaths (<1.0%). (The term “legal intervention” is a classification incorporated into the *International Classification of Diseases, Tenth Revision,* and does not denote the lawfulness or legality of the circumstances surrounding a death caused by law enforcement.) Demographic patterns and circumstances varied by manner of death. The suicide rate was higher among males than among females and was highest among adults aged 35–64 years and non-Hispanic American Indian or Alaska Native (AI/AN) and non-Hispanic White persons. The most common method of injury for suicide was a firearm among males and hanging, strangulation, or suffocation among females. Suicide was most often preceded by a mental health, intimate partner, or physical health problem, or a recent or impending crisis during the previous or upcoming 2 weeks. The homicide rate was highest among persons aged 20–24 years and was higher among males than females. Non-Hispanic Black males experienced the highest homicide rate of any racial or ethnic group. The most common method of injury for homicide was a firearm. When the relationship between a homicide victim and a suspect was known, the suspect was most frequently an acquaintance or friend for male victims and a current or former intimate partner for female victims. Homicides most often were precipitated by an argument or conflict, occurred in conjunction with another crime, or, for female victims, were related to intimate partner violence. Homicide suspects were primarily male and the highest proportion were aged 25–44 years. When race and ethnicity information was known, non-Hispanic Black persons comprised the largest group of suspects overall and among those aged ≤44 years, and non-Hispanic White persons comprised the largest group of suspects among those aged ≥45 years. Almost all legal intervention deaths were experienced by males, and the legal intervention death rate was highest among males aged 30–34 years. Non-Hispanic AI/AN males had the highest legal intervention death rate, followed by non-Hispanic Black males. A firearm was used in the majority of legal intervention deaths. When a specific type of crime was known to have precipitated a legal intervention death, the type of crime was most frequently assault or homicide. The most frequent circumstances reported for legal intervention deaths were use of a weapon by the victim in the incident and a mental health or perceived substance use problem (other than alcohol use). Law enforcement officers who inflicted fatal injuries in the context of legal intervention deaths were primarily males aged 25–44 years. Unintentional firearm deaths were most frequently experienced by males, non-Hispanic White persons, and persons aged 15–24 years. These deaths most often occurred while the shooter was playing with a firearm and most frequently were precipitated by a person unintentionally pulling the trigger or mistakenly thinking that the firearm was unloaded. The rate of deaths of undetermined intent was highest among males, particularly among non-Hispanic Black and non-Hispanic AI/AN males, and among persons aged 45–54 years. Poisoning was the most common method of injury in deaths of undetermined intent, and opioids were detected in approximately 80% of decedents tested for those substances.

**Interpretation:**

This report provides a detailed summary of data from NVDRS on violent deaths that occurred in 2018. The suicide rate was highest among non-Hispanic AI/AN and non-Hispanic White males, and the homicide rate was highest among non-Hispanic Black males. Mental health problems, intimate partner problems, interpersonal conflicts, and acute life stressors were primary circumstances for multiple types of violent death. Circumstances for suspects of homicide varied by age group and included having prior contact with law enforcement and involvement in incidents that were precipitated by another crime, intimate partner violence, and drug dealing or substance use.

**Public Health Action:**

NVDRS data are used to monitor the occurrence of violence-related fatal injuries and assist public health authorities in developing, implementing, and evaluating programs, policies, and practices to reduce and prevent violent deaths. For example, Arizona and Wisconsin used their state-level VDRS data to support suicide prevention efforts within their respective states. Wisconsin VDRS used multiple years of data (2013–2017) to identify important risk and protective factors and subsequently develop a comprehensive suicide prevention plan. Arizona VDRS partners with the Arizona Be Connected Initiative to provide customized community-level data on veteran suicide deaths in Arizona. Similarly, states participating in NVDRS have used their VDRS data to examine intimate partner violence–related deaths to support prevention efforts. For example, data from the South Carolina VDRS were used to examine intimate partner homicides that occurred in South Carolina during 2017. South Carolina VDRS found that 12% of all homicides that occurred in 2017 were intimate partner violence–related, with females accounting for 52% of intimate partner homicide–related victims. These data were shared with domestic violence prevention collaborators in South Carolina to bolster their efforts in reducing intimate partner violence–related deaths. In 2018, NVDRS data included four additional states compared with 2017, providing more comprehensive and actionable violent death information for public health efforts to reduce violent deaths.

## Introduction

In 2018, violence-related injuries led to approximately 68,000 deaths in the United States (*1*). Suicide was the 10th leading cause of death overall in the United States and disproportionately affected young and middle-aged populations. By age group, suicide was the second leading cause of death for persons aged 10–34 years and the fourth leading cause of death for persons aged 35–54 years. During 2018, non-Hispanic American Indian or Alaska Native (AI/AN) and non-Hispanic White males were disproportionately affected by suicide.

In 2018, homicide was the 16th leading cause of death overall in the United States but disproportionately affected young persons (*1*). Homicide was among the five leading causes of death for children aged 1–14 years, was the third leading cause of death for persons aged 15–34 years and was the fifth leading cause of death for persons aged 35–44 years. Young non-Hispanic Black males also were disproportionately affected by homicide. Homicide was the leading cause of death for non-Hispanic Black males aged 15–34 years, the second leading cause of death for those aged 1–9 years, and the third leading cause of death for those aged 10–14 years.

Public health authorities require accurate, timely, and complete surveillance data to better understand and ultimately prevent the occurrence of violent deaths in the United States (*2*,*3*). In 2000, in response to an Institute of Medicine* report noting the need for a national fatal intentional injury surveillance system (*4*), CDC began planning to implement NVDRS (*2*). The goals of NVDRS are to

- collect and analyze timely, high-quality data for monitoring the magnitude and characteristics of violent deaths at national, state, and local levels;
- ensure data are disseminated routinely and expeditiously to public health officials, law enforcement officials, policymakers, and the public;
- ensure data are used to develop, implement, and evaluate programs and strategies that are intended to reduce and prevent violent deaths and injuries at national, state, and local levels; and
- build and strengthen partnerships among organizations and communities at national, state, and local levels to ensure that data are collected and used to reduce and prevent violent deaths and injuries.

NVDRS is a state-based active surveillance system that collects data on the characteristics and circumstances associated with violence-related deaths in participating states, the District of Columbia, and Puerto Rico (*2*). Deaths collected by NVDRS include suicides, homicides, legal intervention deaths (i.e., deaths caused by law enforcement acting in the line of duty and other persons with legal authority to use deadly force, excluding legal executions), unintentional firearm deaths, and deaths of undetermined intent that might have been due to violence.^†^ The term “legal intervention” is a classification incorporated into the *International Classification of Diseases, Tenth Revision* (*ICD-10*) (*5*) and does not denote the lawfulness or legality of the circumstances surrounding a death caused by law enforcement.

Before implementation of NVDRS, single data sources (e.g., death certificates) provided only limited information and few circumstances from which to understand patterns of violent deaths. NVDRS filled this surveillance gap by providing more detailed information. NVDRS is the first system to 1) provide detailed information on circumstances precipitating violent deaths, 2) link multiple source documents so that each incident can contribute to the study of patterns of violent deaths, and 3) link multiple deaths that are related to one another (e.g., multiple homicides, suicide pacts, or homicide followed by suicide of the suspect).

NVDRS data collection began in 2003 with six participating states (Maryland, Massachusetts, New Jersey, Oregon, South Carolina, and Virginia) (Figure). Seven states (Alaska, Colorado, Georgia, North Carolina, Oklahoma, Rhode Island, and Wisconsin) began data collection in 2004, three (Kentucky, New Mexico, and Utah) in 2005, two (Ohio and Michigan) in 2010, and 14 (Arizona, Connecticut, Hawaii, Illinois, Indiana, Iowa, Kansas, Maine, Minnesota, New Hampshire, New York, Pennsylvania, Vermont, and Washington) in 2015. In 2017, eight additional states (Alabama, California, Delaware, Louisiana, Missouri, Nebraska, Nevada, and West Virginia) began data collection, along with the District of Columbia and Puerto Rico.^§^ NVDRS received funding in 2018 for a nationwide expansion that included the remaining 10 states (Arkansas, Florida, Idaho, Mississippi, Montana, North Dakota, South Dakota, Tennessee, Texas, and Wyoming), which began data collection in 2019. CDC now provides NVDRS funding to all 50 states, the District of Columbia, and Puerto Rico. NVDRS data are updated annually and are available to the public through CDC’s Web-based Injury Statistics Query and Reporting System (WISQARS)^¶^ at https://www.cdc.gov/injury/wisqars/nvdrs.html. Case-level NVDRS data are available to interested researchers who meet eligibility requirements via the NVDRS Restricted Access Database (https://www.cdc.gov/violenceprevention/datasources/nvdrs/dataaccess.html).

**FIGURE:**
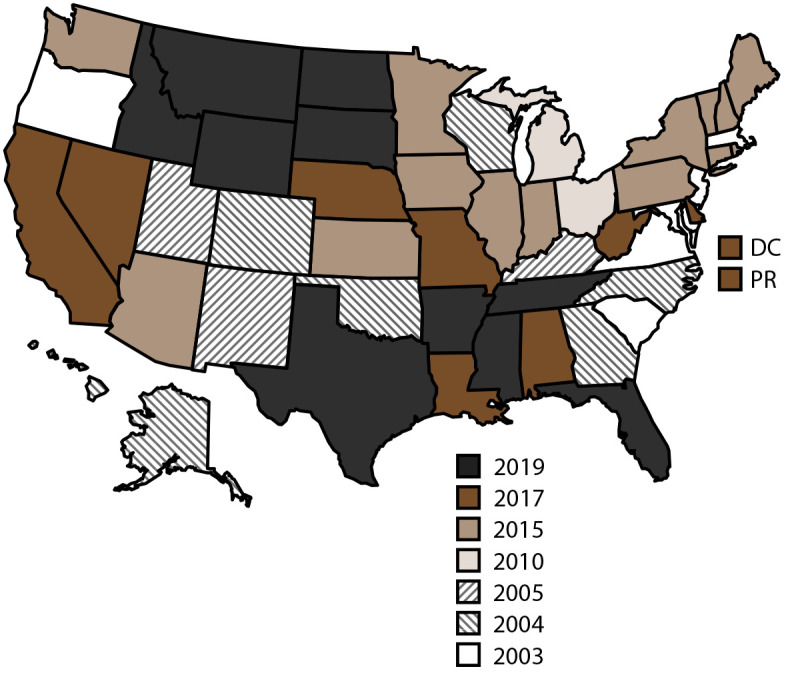
States participating in the National Violent Death Reporting System, by year of initial data collection* — United States and Puerto Rico, 2003–2021 **Abbreviations:** DC = District of Columbia; NVDRS = National Violent Death Reporting System; PR = Puerto Rico. * Map of the United States indicates the year in which the state or territory began collecting data in the National Violent Death Reporting System. California began collecting data for a subset of violent deaths in 2005 but ended data collection in 2009. In 2017, California collected data from death certificates for all NVDRS cases in the state; data for violent deaths that occurred in four counties (Los Angeles, Sacramento, Shasta, and Siskiyou) also include information from coroner or medical examiner reports and law enforcement reports. In 2018, California collected data from death certificates for all violent deaths in the state in 2018 (n = 6,641); data for violent deaths that occurred in 21 counties (Amador, Butte, Fresno, Humboldt, Imperial, Kern, Kings, Lake, Los Angeles, Marin, Mono, Placer, Sacramento, San Benito, San Mateo, San Diego, San Francisco, Shasta, Siskiyou, Ventura, and Yolo) also included information from coroner or medical examiner reports and law enforcement (n = 3,658; 55.1%). Michigan collected data for a subset of violent deaths during 2010–2013 and collected statewide data beginning in 2014. In 2016, Illinois, Pennsylvania, and Washington began collecting data on violent deaths in a subset of counties that represented at least 80% of all violent deaths in their state or in counties where at least 1,800 violent deaths occurred. 2018 data for Illinois are for violent deaths that occurred in 28 counties (Adams, Boone, Champaign, Cook, DuPage, Effingham, Fulton, Kane, Kankakee, Kendall, Lake, Lasalle, Livingston, Logan, McDonough, McHenry, McLean, Macoupin, Madison, Peoria, Perry, Rock Island, St. Clair, Sangamon, Tazewell, Vermillion, Will, and Winnebago). 2018 data for Pennsylvania are for deaths that occurred in 39 counties (Adams, Allegheny, Armstrong, Beaver, Berks, Blair, Bradford, Bucks, Cambria, Carbon, Centre, Chester, Clarion, Clearfield, Clinton, Columbia, Crawford, Dauphin, Delaware, Fayette, Forest, Greene, Indiana, Jefferson, Lackawanna, Lancaster, Lehigh, Luzerne, Monroe, Montgomery, Montour, Northampton, Philadelphia, Schuylkill, Union, Wayne, Westmoreland, Wyoming, and York). In 2018, Washington began collecting statewide data. Beginning in 2019, all 50 U.S. states, the District of Columbia, and Puerto Rico were participating in the system.

This report summarizes NVDRS data on violent deaths that occurred in 39 states, the District of Columbia, and Puerto Rico in 2018. Thirty-six states (Alabama, Alaska, Arizona, Colorado, Connecticut, Delaware, Georgia, Indiana, Iowa, Kansas, Kentucky, Louisiana, Maine, Maryland, Massachusetts, Michigan, Minnesota, Missouri, Nebraska, Nevada, New Hampshire, New Jersey, New Mexico, New York, North Carolina, Ohio, Oklahoma, Oregon, Rhode Island, South Carolina, Utah, Vermont, Virginia, Washington, West Virginia, and Wisconsin) collected statewide data, and three states collected data from a subset of counties in their states (21 California counties, 28 Illinois counties, and 39 Pennsylvania counties). This report highlights information about suspected perpetrators (suspects) of homicides in deaths in which information about the suspect is known, and law enforcement officers who inflicted fatal injuries in legal intervention deaths in which information about the officer is known. Information on suspects can be used to support violence prevention efforts by providing a more complete understanding of the contextual factors related to fatal violence perpetration and the circumstances surrounding these incidents.

## Methods

NVDRS compiles information from three required data sources: death certificates, coroner and medical examiner reports, and law enforcement reports (*2*). Some participating Violent Death Reporting System (VDRS) programs might also collect information from secondary sources (e.g., child fatality review team data, Federal Bureau of Investigation Supplementary Homicide Reports, and crime laboratory data). NVDRS combines information for each death and links deaths that are related (e.g., multiple homicides, homicide followed by suicide, or multiple suicides) into a single incident. The ability to analyze linked data can provide a more comprehensive understanding of violent deaths. Participating VDRS programs use vital statistics death certificate files or coroner or medical examiner reports to identify violent deaths meeting the NVDRS case definition (see Manner of Death). Each VDRS program reports violent deaths of residents that occurred within the state, district, or territory (i.e., resident deaths) and those of nonresidents for whom a fatal injury occurred within the state, district, or territory (i.e., occurrent deaths). When a violent death is identified, NVDRS data abstractors link source documents, link deaths within each incident, code data elements, and write brief narratives of the incident.

In NVDRS, a violent death is defined as a death resulting from the intentional use of physical force or power, threatened or actual, against oneself, another person, or a group or community (*2*). NVDRS collects information on five manners of death: 1) suicide, 2) homicide, 3) legal intervention death, 4) unintentional firearm death, and 5) death of undetermined intent that might have been due to violence (see Manner of Death). NVDRS cases are coded based on *ICD-10* (*5*) or the manner of death assigned by a coroner, medical examiner, or law enforcement officer. Cases are included if they are assigned *ICD-10* codes (Box 1) or a manner of death specified in at least one of the three primary data sources consistent with NVDRS case definitions.

BOX 1 ***International Classification of Diseases, Tenth Revision* (*ICD-10*) codes used in the National Violent Death Reporting System**

**Table:** 

Manner of death	Death ≤1 year after injury	Death >1 year after injury	Death any time after injury
Intentional self-harm (suicide)	X60–X84	Y87.0	U03 (attributable to terrorism)
Assault (homicide)	X85–X99, Y00–Y09	Y87.1	U01, U02 (attributable to terrorism)
Event of undetermined intent	Y10–Y34	Y87.2, Y89.9	Not applicable
Unintentional exposure to inanimate mechanical forces (firearms)	W32–W34	Y86	Not applicable
Legal intervention (excluding executions, Y35.5)	Y35.0–Y35.4, Y35.6, Y35.7	Y89.0	Not applicable

NVDRS is an incident-based system, and all decedents associated with a given incident are grouped in one record. Decisions about whether two or more deaths are related and belong to the same incident are made based on the timing of the injuries rather than on the timing of the deaths. Deaths resulting from injuries that are clearly linked by source documents and occur within 24 hours of each other (see Manner of Death) are considered part of the same incident. Examples of an incident include 1) a single isolated violent death, 2) two or more related homicides (including legal intervention deaths) when the fatal injuries were inflicted <24 hours apart, 3) two or more related suicides or deaths of undetermined intent when the fatal injuries were inflicted <24 hours apart, and 4) a homicide followed by a suicide when both fatal injuries were inflicted <24 hours apart (*6*).

Information collected from each data source is entered into the NVDRS web-based system (*2*). This system streamlines data abstraction by allowing abstractors to enter data from multiple sources into the same incident record. Internal validation checks, hover-over features that define selected fields, and other quality control measures are included. Primacy rules and hierarchal algorithms related to the source documents occur at the local VDRS program level. CDC provides access to the web-based system to each VDRS program. VDRS program personnel are provided ongoing coding training to learn and adhere to CDC guidance regarding the coding of all variables and technical assistance to help increase data quality. Data are transmitted continuously via the web to a CDC-based server. Information abstracted into the system is deidentified at the local VDRS program level.

### Manner of Death

A manner (i.e., intent) of death for each decedent is assigned by a trained abstractor who integrates information from all source documents. The abstractor-assigned manner of death must be consistent with at least one required data source; typically, all source documents are consistent regarding the manner of death. When a discrepancy exists, the abstractor must assign a manner of death on the basis of a preponderance of evidence in the source documents; however, such occurrences are rare (*6*). For example, if two sources report a death as a suicide and a third reports it as a death of undetermined intent, the death is coded as a suicide.

NVDRS data are categorized into five abstractor-assigned manners of death: 1) suicide, 2) homicide, 3) legal intervention death, 4) unintentional firearm death, and 5) death of undetermined intent. The case definitions for each manner of death are described as follows:

- **Suicide.** A suicide is a death of a person aged ≥10 years resulting from the use of force against oneself when a preponderance of evidence indicates that the use of force was intentional. This category also includes the following scenarios: 1) deaths of persons who intended only to injure rather than kill themselves; 2) persons who initially intended to kill themselves but changed their minds and died as a result of the act; 3) deaths associated with risk-taking behavior without clear intent to inflict fatal self-injury but associated with high risk for death (e.g., participating in Russian roulette); 4) suicides that occurred while under the influence of substances taken voluntarily; 5) suicides among decedents with mental health problems that affected their thinking, feelings, or mood (e.g., while experiencing an acute episode of a mental health condition such as schizophrenia or other psychotic conditions, depression, or posttraumatic stress disorder [PTSD]); and 6) suicides involving another person who provided only passive assistance to the decedent (e.g., supplying the means or information needed to complete the act). This category does not include deaths caused by chronic or acute substance use without the intent to die, deaths attributed to autoerotic behavior (e.g., self-strangulation during sexual activity), or assisted suicides (legal or nonlegal). Corresponding *ICD-10* codes included in NVDRS are X60–X84, Y87.0, and U03 (Box 1).
- **Homicide.** A homicide is a death resulting from the use of physical force or power, threatened or actual, against another person, group, or community when a preponderance of evidence indicates that the use of force was intentional. Two special scenarios that CDC’s National Center for Health Statistics (NCHS) regards as homicides are included in the NVDRS case definition: 1) arson with no specified intent to injure someone and 2) a stabbing with intent unspecified. This category also includes the following scenarios: 1) deaths when the suspect intended only to injure rather than kill the victim, 2) deaths resulting from a heart attack induced when the suspect used force or power against the victim, 3) deaths that occurred when a person killed an attacker in self-defense, 4) deaths resulting from a weapon that discharged unintentionally while being used to control or frighten a victim, 5) deaths attributed to child abuse without intent being specified, 6) deaths attributed to an intentional act of neglect by one person against another, 7) deaths of live-born infants that resulted from a direct injury due to violence sustained before birth, and 8) deaths identified as a justifiable homicide when the person committing the homicide was not a law enforcement officer. This category excludes vehicular homicide without intent to injure, unintentional poisoning deaths attributable to illicit or prescription drug overdose even when the person who provided the drugs was charged with homicide, unintentional firearm deaths (a separate category in NVDRS), combat deaths or acts of war, deaths of unborn fetuses, and deaths of infants that resulted indirectly from violence sustained by the mother before birth (e.g., death from prematurity after premature labor brought on by violence). Corresponding *ICD-10* codes included in NVDRS are X85–X99, Y00–Y09, Y87.1, and U01–U02 (Box 1).
- **Legal intervention.** A death from legal intervention is a death in which a person is killed or died as a result of injuries inflicted by a law enforcement officer or other peace officer (i.e., a person with specified legal authority to use deadly force), including military police, while acting in the line of duty. The term “legal intervention” is a classification from *ICD-10* (Y35.0) and does not denote the lawfulness or legality of the circumstances surrounding a death caused by law enforcement. Legal intervention deaths also include a small subset of cases in which force was applied without clear lethal intent (e.g., during restraint or when applying force with a typically nondeadly weapon, such as a Taser) or in which the death occurred while the person was fleeing capture. This category excludes legal executions. Corresponding *ICD-10* codes included in NVDRS are Y35.0–Y35.4, Y35.6, Y35.7, and Y89.0 (Box 1).
- **Unintentional firearm.** An unintentional firearm death is a death resulting from a penetrating injury or gunshot wound from a weapon that uses a powder charge to fire a projectile and for which a preponderance of evidence indicates that the shooting was not directed intentionally at the decedent. Examples include the following: 1) a person who received a self-inflicted wound while playing with a firearm; 2) a person who mistakenly believed a gun was unloaded and shot another person; 3) a child aged <6 years who shot himself or herself or another person; 4) a person who died as a result of a celebratory firing that was not intended to frighten, control, or harm anyone; 5) a person who unintentionally shot himself or herself when using a firearm to frighten, control, or harm another person; 6) a soldier who was shot during a field exercise but not in a combat situation; and 7) an infant who died after birth from an unintentional firearm injury that was sustained in utero. This category excludes injuries caused by unintentionally striking a person with the firearm (e.g., hitting a person on the head with the firearm rather than firing a projectile) and unintentional injuries from nonpowder guns (e.g., BB, pellet, or other compressed air-powered or gas-powered guns). Corresponding *ICD-10* codes included in NVDRS are W32–W34 and Y86 (Box 1).
- **Undetermined intent.** A death of undetermined intent in NVDRS is a death resulting from the use of force or power against oneself or another person for which the evidence indicating one manner of death is no more compelling than evidence indicating another. This category includes coroner or medical examiner rulings where records from data providers indicate that investigators did not find enough evidence to determine whether the injury was intentional (e.g., unclear whether a drug overdose was unintentional or a suicide). Corresponding *ICD-10* codes included in NVDRS are Y10–Y34, Y87.2, and Y89.9 (Box 1).

### Variables Analyzed

NVDRS collects up to approximately 600 unique variables for each death (Boxes 1, 2, and 3). The number of variables recorded for each incident depends on the content and completeness of the source documents. Variables in NVDRS include

BOX 2 **Methods used to inflict injury — National Violent Death Reporting System, 2018**

- Firearm: method that uses a powder charge to fire a projectile from the weapon (excludes BB gun, pellet gun, and compressed air or gas-powered gun)
- Hanging, strangulation, or suffocation (e.g., hanging by the neck, manual strangulation, or plastic bag over the head)
- Poisoning (e.g., fatal ingestion of a street drug, pharmaceutical, carbon monoxide, gas, rat poison, or insecticide)
- Sharp instrument (e.g., knife, razor, machete, or pointed instrument)
- Blunt instrument (e.g., club, bat, rock, or brick)
- Fall: being pushed or jumping
- Motor vehicle (e.g., car, bus, motorcycle, or other transport vehicle)
- Personal weapons (e.g., hands, fists, or feet)
- Drowning: inhalation of liquid (e.g., in bathtub, lake, or other source of water or liquid)
- Fire or burns: inhalation of smoke or the direct effects of fire or chemical burns
- Intentional neglect: starvation, lack of adequate supervision, or withholding of health care
- Other (single method): any method other than those already listed (e.g., electrocution, exposure to environment or weather, or explosives)
- Unknown: method not reported or not known

BOX 3 **Circumstances preceding fatal injury, by manner of death — National Violent Death Reporting System, 2018**

**Suicide/Undetermined Intent**

- Intimate partner problem: decedent was experiencing problems with a current or former intimate partner.
- Suicide of family member or friend: decedent was distraught over, or reacting to, the recent suicide of a family member or friend.
- Other death of family member or friend: decedent was distraught over, or reacting to, the recent nonsuicide death of a family member or friend.
- Physical health problem: decedent was experiencing physical health problems (e.g., a recent cancer diagnosis or chronic pain).
- Job problem: decedent was either experiencing a problem at work or was having a problem with joblessness.
- Recent criminal legal problem: decedent was facing criminal legal problems (e.g., recent or impending arrest or upcoming criminal court date).
- Noncriminal legal problem: decedent was facing civil legal problems (e.g., a child custody or civil lawsuit).
- Financial problem: decedent was experiencing financial problems (e.g., bankruptcy, overwhelming debt, or foreclosure of a home or business).
- Eviction or loss of home: decedent was experiencing a recent or impending eviction or other loss of housing, or the threat of eviction or loss of housing.
- School problem: decedent was experiencing a problem related to school (e.g., poor grades, bullying, social exclusion at school, or performance pressures).
- Traumatic anniversary: the incident occurred on or near the anniversary of a traumatic event in the decedent’s life.
- Exposure to disaster: decedent was exposed to a disaster (e.g., earthquake or bombing).
- Left a suicide note: decedent left a note, e-mail message, video, or other communication indicating intent to die by suicide.
- Disclosed suicidal intent: decedent had recently expressed suicidal feelings to another person with time for that person to intervene.
- Disclosed intent to whom: type of person (e.g., family member or current or former intimate partner) to whom the decedent recently disclosed suicidal thoughts or plans.
- History of suicidal thoughts or plans: decedent had previously expressed suicidal thoughts or plans.
- History of suicide attempt: decedent had previously attempted suicide before the fatal incident.

**Homicide/Legal Intervention**

- Jealousy (lovers’ triangle): jealousy or distress over an intimate partner’s relationship or suspected relationship with another person.
- Stalking: pattern of unwanted harassing or threatening tactics by either the decedent or suspect.
- Prostitution: prostitution or related activity that includes prostitutes, pimps, clients, or others involved in such activity.
- Drug involvement: drug dealing, drug trade, or illicit drug use that is suspected to have played a role in precipitating the incident.
- Brawl: mutual physical fight involving three or more persons.
- Mercy killing: decedent wished to die because of a terminal or hopeless disease or condition, and documentation indicates that the decedent wanted to be killed.
- Victim was a bystander: decedent was not the intended target in the incident (e.g., pedestrian walking past a gang fight).
- Victim was a police officer on duty: decedent was a law enforcement officer killed in the line of duty.
- Victim was an intervener assisting a crime victim: decedent was attempting to assist a crime victim at the time of the incident (e.g., a child attempts to intervene and is killed while trying to assist a parent who is being assaulted).
- Victim used a weapon: decedent used a weapon to attack or defend during the course of the incident.
- Intimate partner violence related: incident is related to conflict between current or former intimate partners; includes the death of an intimate partner or nonintimate partner (e.g., child, parent, friend, or law enforcement officer) killed in an incident that originated in a conflict between intimate partners.
- Hate crime: decedent was selected intentionally because of his or her actual or perceived gender, religion, sexual orientation, race, ethnicity, or disability.
- Mentally ill suspect: suspect’s attack on decedent was believed to be the direct result of a mental health problem (e.g., schizophrenia or other psychotic condition, depression, or PTSD).
- Drive-by shooting: suspect drove near the decedent and fired a weapon while driving.
- Walk-by assault: decedent was killed by a targeted attack (e.g., ambush) where the suspect fled on foot.
- Random violence: decedent was killed in a random act of violence (i.e., an act in which the suspect is not concerned with who is being harmed, just that someone is being harmed).
- Gang related: incident resulted from gang activity or gang rivalry; not used if the decedent was a gang member and the death did not appear to result from gang activity.
- Justifiable self-defense: decedent was killed by a law enforcement officer in the line of duty or by a civilian in legitimate self-defense or in defense of others.
- Intimate partner violence related: incident is related to conflict between current or former intimate partners; includes the death of an intimate partner or nonintimate partner (e.g., child, parent, friend, or law enforcement officer) killed in an incident that originated in a conflict between intimate partners.

**Suspect Information**

- Suspected other substance use by suspect: suspected substance use by the suspect in the hours preceding the incident.
- Suspected alcohol use by suspect: suspected alcohol use by the suspect in the hours preceding the incident.
- Suspect had developmental disability: suspect had developmental disability at time of incident.
- Mentally ill suspect: suspect’s attack on decedent was believed to be the direct result of a mental health problem (e.g., schizophrenia or other psychotic condition, depression, or PTSD).
- Prior contact with law enforcement: suspect had contact with law enforcement in the past 12 months.
- Suspect attempted suicide after incident: suspect attempted suicide (fatally or nonfatally) after the death of the victim.
- Suspect recently released from an institution: suspect injured victim within a month of being released from or admitted to an institutional setting (e.g., jail, hospital, psychiatric hospital).

**All Manners of Death (Except Unintentional Firearm**)

- Current depressed mood: decedent was perceived by self or others to be feeling depressed at the time of death.
- Current diagnosed mental health problem: decedent was identified as having a mental health disorder or syndrome listed in the *Diagnostic and Statistical Manual, Version V* (*DSM-V*), with the exception of alcohol and other substance dependence (these are captured in separate variables).
- Type of mental health diagnosis: identifies the type of *DSM-V* diagnosis reported for the decedent.
- Current mental health treatment: decedent was receiving mental health treatment as evidenced by a current prescription for a psychotropic medication, visit or visits to a mental health professional, or participation in a therapy group within the previous 2 months.
- History of ever being treated for mental health problem: decedent was identified as having ever received mental health treatment.
- Alcohol problem: decedent was perceived by self or others to have a problem with, or to be addicted to, alcohol.
- Substance use problem (excludes alcohol): decedent was perceived by self or others to have a problem with, or be addicted to, a substance other than alcohol.
- Other addiction: decedent was perceived by self or others to have an addiction other than to alcohol or other substance (e.g., gambling or sex).
- Family relationship problem: decedent was experiencing problems with a family member, other than an intimate partner.
- Other relationship problem (nonintimate): decedent was experiencing problems with a friend or associate (other than an intimate partner or family member).
- History of child abuse or neglect: as a child, decedent had history of physical, sexual, or psychological abuse; physical (including medical or dental), emotional, or educational neglect; exposure to a violent environment, or inadequate supervision by a caretaker.
- Caretaker abuse or neglect led to death: decedent was experiencing physical, sexual, or psychological abuse; physical (including medical or dental), emotional, or educational neglect; exposure to a violent environment; or inadequate supervision by a caretaker that led to death.
- Perpetrator of interpersonal violence during previous month: decedent perpetrated interpersonal violence during the previous month.
- Victim of interpersonal violence during previous month: decedent was the target of interpersonal violence during the past month.
- Physical fight (two persons, not a brawl): a physical fight between two individuals that resulted in the death of the decedent, who was either involved in the fight, a bystander, or trying to stop the fight.
- Argument or conflict: a specific argument or disagreement led to the victim’s death.
- Precipitated by another crime: incident occurred as the result of another serious crime.
- Nature of crime: the specific type of other crime that occurred during the incident (e.g., robbery or drug trafficking).
- Crime in progress: another serious crime was in progress at the time of the incident.
- Terrorist attack: decedent was injured in a terrorist attack, leading to death.
- Crisis during previous or upcoming 2 weeks: current crisis or acute precipitating event or events that either occurred during the previous 2 weeks or was impending in the following 2 weeks (e.g., a trial for a criminal offense begins the following week) and appeared to have contributed to the death. Crises typically are associated with specific circumstance variables (e.g., job problem was a crisis, or a financial problem was a crisis).
- Other crisis: a crisis related to a death but not captured by any of the standard circumstances.

**Unintentional Firearm Death**

*Context of Injury*

- Hunting: death occurred any time after leaving home for a hunting trip and before returning home from a hunting trip.
- Target shooting: shooter was aiming for a target and unintentionally hit the decedent; can be at a shooting range or an informal backyard setting (e.g., teenagers shooting at signposts on a fence).
- Loading or unloading gun: gun discharged when the shooter was loading or unloading ammunition.
- Cleaning gun: shooter pulled trigger or gun discharged while cleaning, repairing, assembling, or disassembling gun.
- Showing gun to others: gun was being shown to another person when it discharged, or the trigger was pulled.
- Playing with gun: shooter was playing with a gun when it discharged.
- Celebratory firing: shooter fired gun in celebratory manner (e.g., firing into the air at midnight on New Year’s Eve).
- Other context of injury: shooting occurred during some context other than those already described.

*Mechanism of Injury*

- Unintentionally pulled trigger: shooter unintentionally pulled the trigger (e.g., while grabbing the gun or holding it too tightly).
- Thought gun safety was engaged: shooter thought the safety was on and gun would not discharge.
- Thought unloaded or magazine disengaged: shooter thought the gun was unloaded because the magazine was disengaged.
- Thought gun was unloaded: shooter thought the gun was unloaded for other unspecified reason.
- Bullet ricocheted: bullet ricocheted from its intended target and struck the decedent.
- Gun fired due to defect or malfunction: gun had a defect or malfunctioned as determined by a trained firearm examiner.
- Gun fired while holstering: gun was being replaced or removed from holster or clothing.
- Gun was dropped: gun discharged when it was dropped.
- Gun fired while operating safety or lock: shooter unintentionally fired the gun while operating the safety or lock.
- Gun was mistaken for toy: gun was mistaken for a toy and was fired without the user understanding the danger.
- Other mechanism of injury: shooting occurred as the result of a mechanism not already described.

- manner of death (i.e., the intent to cause death [suicide, homicide, legal intervention, unintentional, and undetermined] of the person on whom a fatal injury was inflicted) (Box 1);
- demographic information (e.g., age, sex, and race and ethnicity) of victims and suspects (if applicable);
- method of injury (i.e., the mechanism used to inflict a fatal injury) (Box 2);
- location, date, and time of injury and death;
- toxicology findings (for decedents who were tested);
- circumstances (i.e., the events that preceded and were identified by investigators as relevant and therefore might have contributed to the infliction of a fatal injury) (Box 3);
- whether the decedent was a victim (i.e., a person who died as a result of a violence-related injury) or both a suspect and a victim (i.e., a person believed to have inflicted a fatal injury on a victim who then was fatally injured, such as the perpetrator of a homicide followed by suicide incident);
- information about any known suspect (i.e., a person or persons believed to have inflicted a fatal injury on a victim);
- incident (i.e., an occurrence in which one or more persons sustained a fatal injury that was linked to a common event or perpetrated by the same suspect or suspects during a 24-hour period); and
- type of incident (i.e., a combination of the manner of death and the number of victims in an incident).

### Circumstances Preceding Death

Circumstances preceding death are defined as the precipitating events that contributed to the infliction of a fatal injury (Box 3). Circumstances are reported on the basis of the content of coroner or medical examiner and law enforcement investigative reports. Certain circumstances are coded to a specific manner of death (e.g., history of suicide attempt is collected for suicides or deaths of undetermined intent); other circumstances are coded across all manners of death (e.g., current diagnosed mental health problem). The data abstractor selects from a list of potential circumstances and is required to code all circumstances that are known to relate to each incident. If circumstances are unknown (e.g., a body was found in the woods with no other details reported), the data abstractor does not endorse circumstances; these deaths are then excluded from the denominator for circumstance values. If either the coroner or medical examiner report or law enforcement report indicates the presence of a circumstance, then the abstractor endorses the circumstance (e.g., if the law enforcement report indicated that a decedent had disclosed an intent to die by suicide, then the circumstance variable “disclosed suicidal intent” is endorsed).

Data abstractors draft two incident narratives that summarize the sequence of events of the incident: one from the perspective of the coroner or medical examiner report and one from the perspective of the law enforcement report. In addition to briefly summarizing the incident (i.e., the who, what, when, where, and why), the narratives provide supporting information on circumstances that the data abstractor indicated and context for understanding the incident, record information and additional detail that cannot be captured elsewhere, and facilitate data quality control checks on the coding of key variables.

In NVDRS, the circumstance variable “intimate partner violence related” identifies cases in which the homicide or legal intervention death was related to immediate or ongoing conflict or violence between current or former intimate partners. In this report, intimate partner violence-related homicides include victims killed by an intimate partner (e.g., current, former, or unspecified spouse, boyfriend, or girlfriend) and those killed during an intimate partner violence–related homicide who were not the intimate partner (e.g., children, other family members, friends, or others who might have intervened in intimate partner violence [e.g., first responders or bystanders]).

### Coding Training and Quality Control

Ongoing coding support for data abstractors is provided by CDC through an electronic help desk, monthly conference calls, annual in-person meetings that include coding training for data abstractors, and regular conference calls with individual VDRS programs. In addition, all data abstractors are invited to participate in monthly coding workgroup calls. VDRS programs can conduct additional abstractor training workshops and activities at their own discretion, including through the use of NVDRS Data Abstractor eLearn Training Modules. An NVDRS coding manual (*6*) with CDC-issued standard guidance on coding criteria and examples for each data element is provided to each VDRS program. Software features that enhance coding reliability include automated validation rules and a hover-over feature containing variable-specific information.

VDRS programs are requested to reabstract a subset of cases using multiple abstractors to identify inconsistencies annually. Each VDRS program’s data quality is also evaluated by CDC. Before the data are released each year, CDC conducts a quality control analysis that involves the review of multiple variables for data inconsistencies, with special focus on abstractor-assigned variables (e.g., method of injury and manner of death). If CDC finds inconsistencies, the VDRS program is notified and asked for a response or correction. VDRS programs must meet CDC standards for completeness of circumstance data to be included in the national dataset. VDRS programs must have circumstance information abstracted from either the coroner or medical examiner report or the law enforcement report for at least 50% of cases. However, VDRS programs often far exceed this requirement. For 2018, a total of 85% of suicides, homicides, and legal intervention deaths in NVDRS had circumstance data from either the coroner or medical examiner report or the law enforcement report. In addition, core variables that represent demographic characteristics (e.g., age, sex, and race and ethnicity) and manners of death were missing or unknown for <0.5% of cases. To ensure the final dataset has no duplicate records, during the data closeout process, NVDRS first identifies any records within VDRS programs that match on a subset of 14 key variables and then asks VDRS programs to review these records to determine if they are true duplicates. In any set of records that are true duplicates, one record is retained and the others are deleted. Next, NVDRS uses SAS (version 9.4; SAS Institute) to search for any instances of duplicates of a unique identification variable associated with each decedent record. As a third and final check for duplicates, the SAS dataset is created with an index that only executes successfully if no duplicates of this identification variable are found.

### Time Frame

VDRS programs are required to begin entering all deaths into the web-based system within 4 months from the date in which the violent death occurred. VDRS programs then have an additional 16 months from the end of the calendar year in which the violent death occurred to complete each incident record. For data collection year 2018 (original completion period through April 2020), VDRS programs were given an additional 3 months to complete incident records because of challenges encountered when deploying NVDRS system updates and delays in data collection caused by the COVID-19 pandemic. Although VDRS programs typically meet timeliness requirements, additional details about an incident occasionally arrive after a deadline has passed. New incidents also might be identified after the deadline (e.g., a death certificate is revised, new evidence is obtained that changes a manner of death, or an *ICD-10* misclassification is corrected to meet the NVDRS case definition). These additional data are incorporated into NVDRS when analysis files are updated in real-time in the web-based system. Five months after the 16-month data collection period for the 2018 data year, case counts increased by <0.1%.

### Inclusion Criteria

The inclusion criteria for violent deaths in this report are as follows: 1) cases met the NVDRS case definition for violent death; 2) cases occurred in participating VDRS states, the District of Columbia, or Puerto Rico in 2018; and 3) at least 50% of cases for each included state, district, or territory had circumstance information collected from the coroner or medical examiner report or law enforcement report. All but one eligible VDRS program that collected data in 2018 (Hawaii) met the completeness threshold for circumstances and were included in the report.

Data for violent deaths occurring in 2018 are reported for 39 states, the District of Columbia, and Puerto Rico. Thirty-six states, the District of Columbia, and Puerto Rico collected data on all violent deaths occurring in their jurisdiction. Three of the 39 states (California, Illinois, and Pennsylvania) collected data from a subset of counties their state. In 2018, the 36 states that collected statewide data, 21 California counties, 28 Illinois counties, 39 Pennsylvania counties, and the District of Columbia accounted for 72.0% of the U.S. population (*7*). 

Of the participating VDRS programs, 36 states (Alabama, Alaska, Arizona, Colorado, Connecticut, Delaware, Georgia, Indiana, Iowa, Kansas, Kentucky, Louisiana, Maine, Maryland, Massachusetts, Michigan, Minnesota, Missouri, Nebraska, Nevada, New Hampshire, New Jersey, New Mexico, New York, North Carolina, Ohio, Oklahoma, Oregon, Rhode Island, South Carolina, Utah, Vermont, Virginia, Washington, West Virginia, and Wisconsin) collected information on all violent deaths that occurred in their states in 2018. In addition, data were collected on all violent deaths that occurred in the District of Columbia and Puerto Rico in 2018. Two states (Illinois and Pennsylvania) joined NVDRS with plans to collect data on violent deaths in a subset of counties that represented at least 80% of all violent deaths in their state or in counties where at least 1,800 violent deaths occurred. In 2018, these states reported data on a subset of counties that represented at least 80% of violent deaths in their state. Data were collected for 28 counties in Illinois that represented 86.0% of the state’s population (Adams, Boone, Champaign, Cook, DuPage, Effingham, Fulton, Kane, Kankakee, Kendall, Lake, Lasalle, Livingston, Logan, McDonough, McHenry, McLean, Macoupin, Madison, Peoria, Perry, Rock Island, St. Clair, Sangamon, Tazewell, Vermillion, Will, and Winnebago) (*7*). In Pennsylvania, data were collected for 39 counties that represented 82.2% of the state’s population (Adams, Allegheny, Armstrong, Beaver, Berks, Blair, Bradford, Bucks, Cambria, Carbon, Centre, Chester, Clarion, Clearfield, Clinton, Columbia, Crawford, Dauphin, Delaware, Fayette, Forest, Greene, Indiana, Jefferson, Lackawanna, Lancaster, Lehigh, Luzerne, Monroe, Montgomery, Montour, Northampton, Philadelphia, Schuylkill, Union, Wayne, Westmoreland, Wyoming, and York) (*7*). California collected data from death certificates for all violent deaths in the state in 2018 (n = 6,641) (Supplementary Table S2, https://stacks.cdc.gov/view/cdc/112767). Data for violent deaths that occurred in 21 counties (Amador, Butte, Fresno, Humboldt, Imperial, Kern, Kings, Lake, Los Angeles, Marin, Mono, Placer, Sacramento, San Benito, San Mateo, San Diego, San Francisco, Shasta, Siskiyou, Ventura, and Yolo) also included information from coroner or medical examiner reports and law enforcement reports and are included throughout the rest of the report (n = 3,658; 55.1%). These 21 counties represented 54.0% of California’s population (*7*). Because <100% of violent deaths were reported, data from California, Illinois, and Pennsylvania are not representative of all violent deaths occurring in these three states.

### Analyses

This report includes data for violent deaths that occurred in 2018 in 39 states, the District of Columbia, and Puerto Rico. VDRS program-level data were received by CDC by the extended date of July 31, 2020; an additional 2-week extension was given to New York because of severe disruptions in data collection caused by the COVID-19 pandemic. All data received by CDC as of August 14, 2020, were consolidated and analyzed. The numbers, percentages, and crude rates are presented in aggregate for all deaths by the abstractor-assigned manner of death. The suicide rate was calculated using denominators among populations aged >10 years. The rates for other manners of death used denominators among populations of all ages. The rates for cells with frequency <20 are not reported because of the instability of those rates. Denominators for the rates for the three states that did not collect statewide data (California, Illinois, and Pennsylvania) correspond to the populations of the counties from which data were collected. The rates could not be calculated for certain variables (e.g., circumstances) because denominators were unknown.

Bridged-race 2018 population estimates were used as denominators in the crude rate calculations for the 36 states that collected statewide data, 21 California counties, 28 Illinois counties, 39 Pennsylvania counties, and the District of Columbia (*8*). For compatible numerators for the rate calculations to be derived, records listing multiple races were recoded to a single race, when possible, using race-bridging methods described by NCHS (https://www.cdc.gov/nchs/nvss/bridged_race.htm) (*9*). The rates specific to race and ethnicity are not available for Puerto Rico because the U.S. Census Bureau estimates for Puerto Rico do not include race or Hispanic origin (*10*). Data for Puerto Rico were analyzed separately. Population estimates by sex and age were used as denominators in the crude rate calculations for Puerto Rico (*11*).

## Results

### Violent Deaths in 39 States and the District of Columbia

For 2018, a total of 39 states and the District of Columbia collected data on 52,773 incidents involving 54,170 deaths (Supplementary Table S1, https://stacks.cdc.gov/view/cdc/112767). Suicide (n = 34,726; 64.1%) accounted for the highest rate of violent deaths (16.8 per 100,000 population aged ≥10 years), followed by homicide (n = 13,441; 24.8%) (5.7 per 100,000 population). Deaths of undetermined intent (n = 4,902; 9.0%), legal intervention deaths (n = 764; 1.4%), and unintentional firearm deaths (n = 337; <1.0%) occurred at lower rates (2.1, 0.3, and 0.1 per 100,000 population, respectively). Deaths by manner that include statewide counts and the rates for California are available (Supplementary Table S2, https://stacks.cdc.gov/view/cdc/112767).

### Suicides

#### Sex, Age Group, and Race and Ethnicity

For 2018, a total of 39 states and the District of Columbia collected data on 34,683 incidents involving 34,726 suicide deaths among persons aged ≥10 years. Overall, the suicide rate was 16.8 per 100,000 population aged ≥10 years (Table 1).

**TABLE 1:** Number, percentage,* and rate^†^ of suicides among persons aged ≥10 years,^§^ by selected demographic characteristics of decedent,^¶^ method used, and location in which injury occurred — National Violent Death Reporting System, 39 states and the District of Columbia, 2018**

Characteristic	Male		Female		Total	
	No. (%)	Rate	No. (%)	Rate	No. (%)	Rate
**Age group (yrs)**						
10–14	280 (1.0)	3.7	152 (2.0)	2.1	**432 (1.2)**	**2.9**
15–19	1,353 (5.0)	17.5	394 (5.2)	5.3	**1,747 (5.0)**	**11.5**
20–24	2,311 (8.5)	28.6	496 (6.5)	6.4	**2,807 (8.1)**	**17.7**
25–29	2,392 (8.8)	27.6	572 (7.5)	6.9	**2,965 (8.5)**	**17.4**
30–34	2,224 (8.2)	27.6	623 (8.2)	7.9	**2,848 (8.2)**	**17.9**
35–44	4,183 (15.4)	28.4	1,259 (16.5)	8.5	**5,442 (15.7)**	**18.4**
45–54	4,480 (16.5)	30.2	1,530 (20.1)	10.0	**6,010 (17.3)**	**20.0**
55–64	4,678 (17.3)	31.4	1,504 (19.7)	9.4	**6,182 (17.8)**	**20.0**
65–74	2,766 (10.2)	26.9	738 (9.7)	6.3	**3,504 (10.1)**	**15.9**
75–84	1,691 (6.2)	35.3	243 (3.2)	3.9	**1,934 (5.6)**	**17.5**
≥85	737 (2.7)	44.4	104 (1.4)	3.4	**841 (2.4)**	**17.7**
Unknown	13 (<1.0)	—^††^	1 (<1.0)	—	**14 (<1.0)**	**—**
**Race/Ethnicity**						
White, non-Hispanic	21,960 (81.0)	32.7	6,124 (80.4)	8.8	**28,086 (80.9)**	**20.5**
Black, non-Hispanic	1,925 (7.1)	14.9	509 (6.7)	3.5	**2,434 (7.0)**	**8.9**
American Indian or Alaska Native, non-Hispanic	413 (1.5)	45.6	124 (1.6)	12.9	**537 (1.5)**	**28.8**
Asian or Pacific Islander, non-Hispanic	749 (2.8)	12.7	328 (4.3)	5.0	**1,077 (3.1)**	**8.7**
Hispanic^§§^	1,979 (7.3)	13.7	507 (6.7)	3.6	**2,486 (7.2)**	**8.7**
Other race or ethnicity	64 (<1.0)	—	23 (<1.0)	—	**87 (<1.0)**	**—**
Unknown	18 (<1.0)	—	1 (<1.0)	—	**19 (<1.0)**	**—**
**Method**						
Firearm	14,493 (53.5)	14.3	2,234 (29.3)	2.1	**16,727 (48.2)**	**8.1**
Hanging, strangulation, or suffocation	7,873 (29.0)	7.8	2,360 (31.0)	2.2	**10,235 (29.5)**	**4.9**
Poisoning	2,078 (7.7)	2.1	2,230 (29.3)	2.1	**4,308 (12.4)**	**2.1**
Fall	639 (2.4)	0.6	236 (3.1)	0.2	**875 (2.5)**	**0.4**
Sharp instrument	548 (2.0)	0.5	124 (1.6)	0.1	**672 (1.9)**	**0.3**
Motor vehicle (e.g., bus, motorcycle, or other transport vehicle)	445 (1.6)	0.4	119 (1.6)	0.1	**564 (1.6)**	**0.3**
Drowning	206 (<1.0)	0.2	121 (1.6)	0.1	**327 (<1.0)**	**0.2**
Fire or burns	106 (<1.0)	0.1	40 (<1.0)	<0.1	**146 (<1.0)**	**<0.1**
Blunt instrument	34 (<1.0)	<0.1	11 (<1.0)	—	**45 (<1.0)**	**<0.1**
Other (e.g., Taser, electrocution, nail gun, intentional neglect, or personal weapon)	39 (<1.0)	—	14 (<1.0)	—	**53 (<1.0)**	**—**
Unknown	647 (2.4)	—	127 (1.7)	—	**774 (2.2)**	**—**
**Location**						
House or apartment	18,958 (69.9)	18.7	5,962 (78.3)	5.6	**24,921 (71.8)**	**12.0**
Motor vehicle	1,474 (5.4)	1.5	309 (4.1)	0.3	**1,784 (5.1)**	**0.9**
Natural area	1,376 (5.1)	1.4	262 (3.4)	0.3	**1,638 (4.7)**	**0.8**
Hotel or motel	562 (2.1)	0.6	225 (3.0)	0.2	**787 (2.3)**	**0.4**
Street or highway	674 (2.5)	0.7	105 (1.4)	0.1	**779 (2.2)**	**0.4**
Park, playground, or sports or athletic area	468 (1.7)	0.5	62 (<1.0)	<0.1	**530 (1.5)**	**0.3**
Parking lot, public garage, or public transport	431 (1.6)	0.4	75 (<1.0)	<0.1	**506 (1.5)**	**0.2**
Jail or prison	432 (1.6)	0.4	47 (<1.0)	<0.1	**479 (1.4)**	**0.2**
Bridge	229 (<1.0)	0.2	69 (<1.0)	<0.1	**298 (<1.0)**	**0.1**
Railroad track	179 (<1.0)	0.2	60 (<1.0)	<0.1	**239 (<1.0)**	**0.1**
Commercial or retail area	196 (<1.0)	0.2	27 (<1.0)	<0.1	**223 (<1.0)**	**0.1**
Supervised residential facility	109 (<1.0)	0.1	38 (<1.0)	<0.1	**147 (<1.0)**	**<0.1**
Hospital or medical facility	100 (<1.0)	0.1	27 (<1.0)	<0.1	**127 (<1.0)**	**<0.1**
Cemetery, graveyard, or other burial ground	84 (<1.0)	<0.1	13 (<1.0)	—	**97 (<1.0)**	**<0.1**
Industrial or construction area	87 (<1.0)	<0.1	8 (<1.0)	—	**95 (<1.0)**	**<0.1**
Preschool, school, college, or school bus	79 (<1.0)	<0.1	16 (<1.0)	—	**95 (<1.0)**	**<0.1**
Farm	89 (<1.0)	<0.1	6 (<1.0)	—	**95 (<1.0)**	**<0.1**
Other location^¶¶^	474 (1.7)	—	67 (<1.0)	—	**541 (1.6)**	**—**
Unknown	1,107 (4.1)	—	238 (3.1)	—	**1,345 (3.9)**	**—**
**Total**	**27,108 (100.0)**	**26.8**	**7,616 (100.0)**	**7.2**	**34,726 (100.0)**	**16.8**

* Percentages might not total 100% due to rounding.

^†^ Per 100,000 population.

^§^ Suicide is not reported for decedents aged <10 years, as per standard in the suicide prevention literature. Denominators for suicide rates represent the total population aged ≥10 years.

^¶^ Sex was unknown for two decedents.

** Data for all violent deaths were collected in 36 states (Alabama, Alaska, Arizona, California, Colorado, Connecticut, Delaware, Georgia, Illinois, Indiana, Iowa, Kansas, Kentucky, Louisiana, Maine, Maryland, Massachusetts, Michigan, Minnesota, Missouri, Nebraska, Nevada, New Hampshire, New Jersey, New Mexico, New York, North Carolina, Ohio, Oklahoma, Oregon, Pennsylvania, Rhode Island, South Carolina, Utah, Vermont, Virginia, Washington, West Virginia, and Wisconsin), and the District of Columbia. Three states (California, Illinois, and Pennsylvania) collected data from a subset of counties in their state. Data for violent deaths that occurred in Illinois include 28 counties that represent 86% of the state’s population (Adams, Boone, Champaign, Cook, DuPage, Effingham, Fulton, Kane, Kankakee, Kendall, Lake, Lasalle, Livingston, Logan, McDonough, McHenry, McLean, Macoupin, Madison, Peoria, Perry, Rock Island, St. Clair, Sangamon, Tazewell, Vermillion, Will, and Winnebago). Data for violent deaths that occurred in Pennsylvania include 39 counties that represent 82.2% of the state’s population (Adams, Allegheny, Armstrong, Beaver, Berks, Blair, Bradford, Bucks, Cambria, Carbon, Centre, Chester, Clarion, Clearfield, Clinton, Columbia, Crawford, Dauphin, Delaware, Fayette, Forest, Greene, Indiana, Jefferson, Lackawanna, Lancaster, Lehigh, Luzerne, Monroe, Montgomery, Montour, Northampton, Philadelphia, Schuylkill, Union, Wayne, Westmoreland, Wyoming, and York). Data for violent deaths that occurred in California include 21 counties that represent 54% of the state’s population (Amador, Butte, Fresno, Humboldt, Imperial, Kern, Kings, Lake, Los Angeles, Marin, Mono, Placer, Sacramento, San Benito, San Diego, San Francisco, San Mateo, Shasta, Siskiyou, Ventura, and Yolo). Denominators for the rates for these three states (California, Illinois, and Pennsylvania) represent only the populations of the counties from which the data were collected.

^††^ Rate is not reported when the number of decedents is <20 or when the characteristic response is “other” or “unknown.”

^§§^ Includes persons of any race.

^¶¶^ Other location includes (in descending order) office building; abandoned house, building, or warehouse; synagogue, church, or temple; bar or nightclub; and other unspecified location.

The suicide rate for males (26.8 per 100,000 population) was 3.7 times the rate for females (7.2 per 100,000 population) (Table 1). The suicide rate for males ranged from 1.8 to 13.1 times the rate for females across age groups and 2.5 to 4.3 times the rate for females across racial and ethnic groups. Adults aged 45–54 years (20.0 per 100,000 population), 55–64 years (20.0 per 100,000 population), and 35–44 years (18.4 per 100,000 population) had the highest rates of suicide across age groups. Non-Hispanic White persons accounted for most (80.9%) of suicides; however, non-Hispanic AI/AN persons had the highest rate of suicide (28.8 per 100,000 population) among racial and ethnic groups.

Among male suicide decedents, approximately one half (49.2%) were aged 35–64 years (Table 1). Males aged ≥85 years had the highest rate of suicide (44.4 per 100,000 population), followed by males aged 75–84 years (35.3 per 100,000 population) and those aged 55–64 years (31.4 per 100,000 population). Non-Hispanic AI/AN males had the highest rate of suicide (45.6 per 100,000 population), followed by non-Hispanic White males (32.7 per 100,000 population). The rate of suicide for non-Hispanic AI/AN males was 3.6 times the rate for males with the lowest rate, Asians and Pacific Islanders (12.7 per 100,000 population). The suicide rate for non-Hispanic Black males was 14.9 per 100,000 population and 13.7 per 100,000 population for Hispanic males.

Among female suicide decedents, those aged 35–64 years accounted for 56.3% of suicides (Table 1). Females aged 45–54 years had the highest rate of suicide (10.0 per 100,000 population). The suicide rate was highest among non-Hispanic AI/AN females (12.9 per 100,000 population), followed by non-Hispanic White (8.8 per 100,000 population), Asian or Pacific Islander (5.0 per 100,000 population), Hispanic (3.6 per 100,000 population), and non-Hispanic Black (3.5 per 100,000 population) females. The suicide rate for non-Hispanic AI/AN females was 3.7 times the rate for females with the lowest rates (non-Hispanic Black females).

#### Method and Location of Injury

A firearm was used in approximately one half (48.2%) of suicides, followed by hanging, strangulation, and suffocation (29.5%) and poisoning (12.4%) (rates of 8.1, 4.9, and 2.1 per 100,000 population, respectively) (Table 1). Among males, the most common method of injury was a firearm (53.5%), followed by hanging, strangulation, or suffocation (29.0%). Among females, hanging, strangulation, or suffocation (31.0%) was the most common method of injury, and poisoning or a firearm were used in equal proportions (29.3%). Among all suicide decedents, the most common location of suicide was a house or apartment (71.8%), followed by a motor vehicle (5.1%), a natural area (4.7%), a hotel or motel (2.3%), and a street or highway (2.2%).

#### Toxicology Results of Decedent

Toxicology tests for alcohol were conducted for 52.3% of suicide decedents (Table 2). Among those with positive results for alcohol (39.8%), 64.0% had a blood alcohol concentration (BAC) ≥0.08 g/dL (Blood alcohol concentration of ≥0.08 g/dL is over the legal limit in all states and the District of Columbia and is used as the standard for intoxication). Tests were conducted for amphetamines, antidepressants, benzodiazepines, cocaine, marijuana, and opioids for 41.3%, 28.2%, 41.1%, 41.8%, 35.4%, and 43.8% of decedents, respectively. Results for opioids (including illicit and prescription) were positive in 22.7% of decedents tested for these substances. Positive results were found for 13.9% of decedents tested for amphetamines, 7.2% of those tested for cocaine, and 23.6% of those tested for marijuana. Of those tested for antidepressants, 35.9% had positive results at the time of death, and 24.6% of those tested for benzodiazepines had positive results. Carbon monoxide was tested for a substantially smaller proportion of decedents (5.9%) but was identified in one third of those decedents (33.5%).

**TABLE 2:** Number* and percentage of suicide decedents tested for alcohol and drugs whose results were positive,^†^ by toxicology variable — National Violent Death Reporting System, 39 states and the District of Columbia, 2018^§^

Toxicology variable	Tested	Positive
	No. (%)	No. (%)
Blood alcohol concentration^¶^	18,179 (52.3)	7,240 (39.8)
Alcohol <0.08 g/dL		2,003 (27.7)
Alcohol ≥0.08 g/dL		4,636 (64.0)
Alcohol positive — level unknown		601 (8.3)
Amphetamines	14,328 (41.3)	1,996 (13.9)
Anticonvulsants	7,668 (22.1)	1,180 (15.4)
Antidepressants	9,793 (28.2)	3,516 (35.9)
Antipsychotics	7,516 (21.6)	842 (11.2)
Barbiturates	12,248 (35.3)	260 (2.1)
Benzodiazepines	14,288 (41.1)	3,513 (24.6)
Carbon monoxide	2,041 (5.9)	684 (33.5)
Cocaine	14,510 (41.8)	1,039 (7.2)
Marijuana	12,297 (35.4)	2,896 (23.6)
Muscle relaxant	7,853 (22.6)	490 (6.2)
Opioids	15,210 (43.8)	3,449 (22.7)
Other drugs or substances**	7,583 (21.8)	6,571 (86.7)

* Number of suicide decedents = 34,726.

^†^ Percentage is of decedents tested for toxicology. Denominator for the percentage positive is the percentage tested.

^§^ Data for all violent deaths were collected in 36 states (Alabama, Alaska, Arizona, California, Colorado, Connecticut, Delaware, Georgia, Illinois, Indiana, Iowa, Kansas, Kentucky, Louisiana, Maine, Maryland, Massachusetts, Michigan, Minnesota, Missouri, Nebraska, Nevada, New Hampshire, New Jersey, New Mexico, New York, North Carolina, Ohio, Oklahoma, Oregon, Pennsylvania, Rhode Island, South Carolina, Utah, Vermont, Virginia, Washington, West Virginia, and Wisconsin), and the District of Columbia. Three states (California, Illinois, and Pennsylvania) collected data from a subset of counties in their state. Data for violent deaths that occurred in Illinois include 28 counties that represent 86% of the state’s population (Adams, Boone, Champaign, Cook, DuPage, Effingham, Fulton, Kane, Kankakee, Kendall, Lake, Lasalle, Livingston, Logan, McDonough, McHenry, McLean, Macoupin, Madison, Peoria, Perry, Rock Island, St. Clair, Sangamon, Tazewell, Vermillion, Will, and Winnebago). Data for violent deaths that occurred in Pennsylvania include 39 counties that represent 82.2% of the state’s population (Adams, Allegheny, Armstrong, Beaver, Berks, Blair, Bradford, Bucks, Cambria, Carbon, Centre, Chester, Clarion, Clearfield, Clinton, Columbia, Crawford, Dauphin, Delaware, Fayette, Forest, Greene, Indiana, Jefferson, Lackawanna, Lancaster, Lehigh, Luzerne, Monroe, Montgomery, Montour, Northampton, Philadelphia, Schuylkill, Union, Wayne, Westmoreland, Wyoming, and York). Data for violent deaths that occurred in California include 21 counties that represent 54% of the state’s population (Amador, Butte, Fresno, Humboldt, Imperial, Kern, Kings, Lake, Los Angeles, Marin, Mono, Placer, Sacramento, San Benito, San Diego, San Francisco, San Mateo, Shasta, Siskiyou, Ventura, and Yolo). Denominators for the rates for these three states (California, Illinois, and Pennsylvania) represent only the populations of the counties from which the data were collected.

^¶^ Blood alcohol concentration of ≥0.08 g/dL is greater than the legal limit in all states and the District of Columbia and is used as the standard for intoxication.

** Other drugs or substances indicated whether any results were positive; levels for these drugs or substances are not measured.

#### Precipitating Circumstances

Circumstances were identified in 30,668 (88.3%) of suicides (Table 3). Overall, a mental health problem was the most common circumstance, with approximately one half (49.7%) of decedents having had a current diagnosed mental health problem and 34.3% experiencing a depressed mood at the time of death. Among the 15,233 decedents with a current diagnosed mental health problem, depression or dysthymia (75.0%), anxiety disorder (19.9%), and bipolar disorder (14.9%) were the most common diagnoses. Among suicide decedents, 26.1% were receiving mental health treatment at the time of death. Alcohol use problems were reported for 18.9% of suicide decedents, and other nonalcohol–related substance use problems were reported for 17.2% of suicide decedents (Table 3).

**TABLE 3:** Number* and percentage^†^ of suicides among persons aged ≥10 years,^§^ by decedent’s sex and precipitating circumstance — National Violent Death Reporting System, 39 states and the District of Columbia, 2018^¶^

Precipitating circumstance	Male	Female	Total
	No. (%)	No. (%)	No. (%)
**Mental health or substance use**			
Current diagnosed mental health problem**	10,736 (45.3)	4,495 (64.7)	**15,233 (49.7)**
Depression or dysthymia	7,972 (74.3)	3,457 (76.9)	**11,431 (75.0)**
Anxiety disorder	1,905 (17.7)	1,126 (25.1)	**3,032 (19.9)**
Bipolar disorder	1,388 (12.9)	877 (19.5)	**2,265 (14.9)**
Schizophrenia	690 (6.4)	219 (4.9)	**909 (6.0)**
PTSD	611 (5.7)	187 (4.2)	**798 (5.2)**
ADD/ADHD	379 (3.5)	68 (1.5)	**447 (2.9)**
OCD	70 (<1.0)	21 (<1.0)	**91 (<1.0)**
Eating disorder	9 (<1.0)	29 (<1.0)	**38 (<1.0)**
Other	690 (6.4)	222 (4.9)	**912 (6.0)**
Unknown	815 (7.6)	356 (7.9)	**1,171 (7.7)**
History of ever being treated for a mental health problem	7,608 (32.1)	3,461 (49.8)	**11,070 (36.1)**
Current depressed mood	8,127 (34.3)	2,384 (34.3)	**10,511 (34.3)**
Current mental health treatment	5,311 (22.4)	2,679 (38.6)	**7,991 (26.1)**
Alcohol problem	4,690 (19.8)	1,099 (15.8)	**5,789 (18.9)**
Substance use problem (excludes alcohol)	4,034 (17.0)	1,249 (18.0)	**5,283 (17.2)**
Other addiction (e.g., gambling or sexual)	158 (<1.0)	42 (<1.0)	**200 (<1.0)**
**Interpersonal**			
Intimate partner problem	6,621 (27.9)	1,655 (23.8)	**8,277 (27.0)**
Family relationship problem	2,027 (8.5)	793 (11.4)	**2,820 (9.2)**
Other death of family member or friend	1,523 (6.4)	543 (7.8)	**2,066 (6.7)**
Suicide of family member or friend	582 (2.5)	236 (3.4)	**818 (2.7)**
Perpetrator of interpersonal violence during past month	648 (2.7)	59 (<1.0)	**707 (2.3)**
Other relationship problem (nonintimate)	499 (2.1)	132 (1.9)	**631 (2.1)**
Victim of interpersonal violence during past month	61 (<1.0)	77 (1.1)	**138 (<1.0)**
**Life stressor**			
Crisis during previous or upcoming 2 weeks	7,583 (32.0)	1,835 (26.4)	**9,419 (30.7)**
Physical health problem	5,121 (21.6)	1,397 (20.1)	**6,518 (21.3)**
Argument or conflict	3,753 (15.8)	1,123 (16.2)	**4,876 (15.9)**
Job problem	2,447 (10.3)	417 (6.0)	**2,864 (9.3)**
Financial problem	2,187 (9.2)	505 (7.3)	**2,693 (8.8)**
Recent criminal legal problem	2,176 (9.2)	233 (3.4)	**2,409 (7.9)**
Eviction or loss of home	864 (3.6)	262 (3.8)	**1,127 (3.7)**
Non-criminal legal problem	874 (3.7)	238 (3.4)	**1,112 (3.6)**
School problem	375 (1.6)	123 (1.8)	**498 (1.6)**
History of child abuse or neglect	223 (<1.0)	141 (2.0)	**364 (1.2)**
Physical fight (two persons, not a brawl)	250 (1.1)	42 (<1.0)	**292 (<1.0)**
Traumatic anniversary	151 (<1.0)	67 (<1.0)	**218 (<1.0)**
Exposure to disaster	58 (<1.0)	4 (<1.0)	**62 (<1.0)**
Caretaker abuse or neglect led to suicide	15 (<1.0)	15 (<1.0)	**30 (<1.0)**
**Crime and criminal activity**			
Precipitated by another crime	1,030 (4.3)	92 (1.3)	**1,122 (3.7)**
Crime in progress^††^	348 (33.8)	27 (29.3)	**375 (33.4)**
**Suicide event**			
History of suicidal thoughts or plans	8,042 (33.9)	2,692 (38.8)	**10,735 (35.0)**
Left a suicide note	7,471 (31.5)	2,765 (39.8)	**10,238 (33.4)**
History of suicide attempt(s)	3,895 (16.4)	2,321 (33.4)	**6,217 (20.3)**
**Suicide disclosure**			
Disclosed suicidal intent^§§^	5,759 (24.3)	1,635 (23.5)	**7,395 (24.1)**
To previous or current intimate partner	2,246 (39.0)	544 (33.3)	**2,790 (37.7)**
To other family member	1,702 (29.5)	528 (32.3)	**2,230 (30.1)**
To friend or colleague	687 (11.9)	218 (13.3)	**905 (12.2)**
To health care worker	238 (4.1)	88 (5.4)	**327 (4.4)**
To neighbor	65 (1.1)	24 (1.5)	**89 (1.2)**
To other person	492 (8.5)	118 (7.2)	**610 (8.2)**
Unknown	332 (5.8)	115 (7.0)	**447 (6.0)**
**Total^¶¶^**	**23,723 (87.5)**	**6,943 (91.2)**	**30,668 (88.3)**

**Abbreviations:** ADD/ADHD = attention deficit disorder/attention deficit hyperactivity disorder; OCD = obsessive-compulsive disorder; PTSD = posttraumatic stress disorder.

* Includes suicides with one or more precipitating circumstances. More than one circumstance could have been present per decedent.

^†^ Denominator includes those suicides with one or more precipitating circumstances. The sums of percentages in columns exceed 100% because more than one circumstance could have been present per decedent.

^§^ Suicide is not reported for decedents aged <10 years, as per standard in the suicide prevention literature.

^¶^ Data for all violent deaths were collected in 36 states (Alabama, Alaska, Arizona, California, Colorado, Connecticut, Delaware, Georgia, Illinois, Indiana, Iowa, Kansas, Kentucky, Louisiana, Maine, Maryland, Massachusetts, Michigan, Minnesota, Missouri, Nebraska, Nevada, New Hampshire, New Jersey, New Mexico, New York, North Carolina, Ohio, Oklahoma, Oregon, Pennsylvania, Rhode Island, South Carolina, Utah, Vermont, Virginia, Washington, West Virginia, and Wisconsin), and the District of Columbia. Three states (California, Illinois, and Pennsylvania) collected data from a subset of counties in their state. Data for violent deaths that occurred in Illinois include 28 counties that represent 86% of the state’s population (Adams, Boone, Champaign, Cook, DuPage, Effingham, Fulton, Kane, Kankakee, Kendall, Lake, Lasalle, Livingston, Logan, McDonough, McHenry, McLean, Macoupin, Madison, Peoria, Perry, Rock Island, St. Clair, Sangamon, Tazewell, Vermillion, Will, and Winnebago). Data for violent deaths that occurred in Pennsylvania include 39 counties that represent 82.2% of the state’s population (Adams, Allegheny, Armstrong, Beaver, Berks, Blair, Bradford, Bucks, Cambria, Carbon, Centre, Chester, Clarion, Clearfield, Clinton, Columbia, Crawford, Dauphin, Delaware, Fayette, Forest, Greene, Indiana, Jefferson, Lackawanna, Lancaster, Lehigh, Luzerne, Monroe, Montgomery, Montour, Northampton, Philadelphia, Schuylkill, Union, Wayne, Westmoreland, Wyoming, and York). Data for violent deaths that occurred in California include 21 counties that represent 54% of the state’s population (Amador, Butte, Fresno, Humboldt, Imperial, Kern, Kings, Lake, Los Angeles, Marin, Mono, Placer, Sacramento, San Benito, San Diego, San Francisco, San Mateo, Shasta, Siskiyou, Ventura, and Yolo). Denominators for the rates for these three states (California, Illinois, and Pennsylvania) represent only the populations of the counties from which the data were collected.

** Includes decedents with one or more diagnosed current mental health problems; therefore, sums of percentages for the diagnosed conditions exceed 100%. Denominator includes the number of decedents with one or more current diagnosed mental health problems.

^††^ Denominator includes those decedents involved in an incident that was precipitated by another crime.

^§§^ Denominator includes decedents who disclosed intent.

^¶¶^ Circumstances were unknown for 4,058 decedents (3,385 males and 673 females); total number of suicide decedents = 34,726 (27,108 males, 7,616 females, and two unknown).

The most commonly reported interpersonal or life stressor precipitating circumstances were a recent or impending crisis during the previous or upcoming 2 weeks (30.7%), an intimate partner problem (27.0%), a physical health problem (21.3%), and an argument or conflict (15.9%). Among other circumstances related to the suicide, one third (33.4%) of decedents left a suicide note, 35.0% had a history of suicidal thoughts or plans, 20.3% had a history of previous suicide attempts, and 24.1% had disclosed suicidal intent to another person. Of those who disclosed intent, the greatest proportion of disclosures was to a previous or current intimate partner (37.7%), followed by a family member other than an intimate partner (30.1%) and a friend or colleague (12.2%).

A larger percentage of female decedents (64.7%) had a current diagnosed mental health problem than did male decedents (45.3%) (Table 3). Male and female suicide decedents had the same percentages of depressed mood at the time of death (34.3%). A larger percentage of female decedents (38.6%) than male decedents (22.4%) were known to have been receiving mental health treatment at the time of death. Suicide events, including leaving a suicide note, history of suicidal thoughts or plans, and history of suicide attempts, occurred more frequently and at higher rates among females than males.

### Homicides

#### Sex, Age Group, and Race and Ethnicity

For 2018, a total of 39 states and the District of Columbia collected data on 12,693 incidents involving 13,441 homicide deaths. Overall, the homicide rate was 5.7 per 100,000 population (Table 4).

**TABLE 4:** Number, percentage,* and rate^†^ of homicides, by selected demographic characteristics of decedent, method used, location in which injury occurred, and victim-suspect relationship^§^ — National Violent Death Reporting System, 39 states and the District of Columbia, 2018^¶^

Characteristic	Male		Female		Total	
	No. (%)	Rate	No. (%)	Rate	No. (%)	Rate
**Age group (yrs)**						
<1	115 (1.1)	8.2	84 (3.0)	6.3	**199 (1.5)**	**7.3**
1–4	149 (1.4)	2.6	105 (3.7)	1.9	**254 (1.9)**	**2.2**
5–9	44 (<1.0)	0.6	32 (1.1)	0.5	**76 (<1.0)**	**0.5**
10–14	56 (<1.0)	0.7	43 (1.5)	0.6	**99 (<1.0)**	**0.7**
15–19	1,022 (9.6)	13.2	182 (6.4)	2.5	**1,204 (9.0)**	**8.0**
20–24	1,764 (16.6)	21.8	317 (11.2)	4.1	**2,081 (15.5)**	**13.2**
25–29	1,840 (17.3)	21.3	326 (11.5)	3.9	**2,166 (16.1)**	**12.7**
30–34	1,344 (12.7)	16.7	276 (9.7)	3.5	**1,620 (12.1)**	**10.2**
35–44	1,925 (18.1)	13.1	470 (16.6)	3.2	**2,395 (17.8)**	**8.1**
45–54	1,124 (10.6)	7.6	365 (12.9)	2.4	**1,489 (11.1)**	**4.9**
55–64	739 (7.0)	5.0	277 (9.8)	1.7	**1,016 (7.6)**	**3.3**
65–74	328 (3.1)	3.2	194 (6.9)	1.7	**522 (3.9)**	**2.4**
75–84	112 (1.1)	2.3	106 (3.7)	1.7	**218 (1.6)**	**2.0**
≥85	45 (<1.0)	2.7	54 (1.9)	1.8	**99 (<1.0)**	**2.1**
Unknown	3 (<1.0)	—**	0 (0)	—	**3 (<1.0)**	—
**Race/Ethnicity**						
White, non-Hispanic	2,489 (23.5)	3.3	1,317 (46.5)	1.7	**3,806 (28.3)**	**2.5**
Black, non-Hispanic	6,218 (58.6)	40.9	1,045 (36.9)	6.3	**7,263 (54.0)**	**22.8**
American Indian or Alaska Native, non-Hispanic	194 (1.8)	18.2	59 (2.1)	5.3	**253 (1.9)**	**11.6**
Asian or Pacific Islander, non-Hispanic	158 (1.5)	2.4	73 (2.6)	1.0	**231 (1.7)**	**1.6**
Hispanic^††^	1,512 (14.3)	8.6	329 (11.6)	1.9	**1,841 (13.7)**	**5.3**
Other race or ethnicity	34 (<1.0)	—	6 (<1.0)	—	**40 (<1.0)**	—
Unknown	5 (<1.0)	—	2 (<1.0)	—	**7 (<1.0)**	—
**Method**						
Firearm	8,035 (75.7)	6.9	1,569 (55.4)	1.3	**9,604 (71.5)**	**4.1**
Sharp instrument	983 (9.3)	0.9	438 (15.5)	0.4	**1,421 (10.6)**	**0.6**
Blunt instrument	373 (3.5)	0.3	197 (7.0)	0.2	**570 (4.2)**	**0.2**
Personal weapons (e.g., hands, feet, or fists)	366 (3.4)	0.3	139 (4.9)	0.1	**505 (3.8)**	**0.2**
Hanging, strangulation, or suffocation	120 (1.1)	0.1	188 (6.6)	0.2	**308 (2.3)**	**0.1**
Motor vehicle (e.g., bus, motorcycle, or other transport vehicle)	89 (<1.0)	<0.1	36 (1.3)	<0.1	**125 (<1.0)**	**<0.1**
Fire or burns	35 (<1.0)	<0.1	36 (1.3)	<0.1	**71 (<1.0)**	**<0.1**
Poisoning	42 (<1.0)	<0.1	23 (<1.0)	<0.1	**65 (<1.0)**	**<0.1**
Intentional neglect	25 (<1.0)	<0.1	26 (<1.0)	<0.1	**51 (<1.0)**	**<0.1**
Fall	30 (<1.0)	<0.1	8 (<1.0)	—	**38 (<1.0)**	**<0.1**
Shaking (e.g., shaken baby syndrome)	17 (<1.0)	—	12 (<1.0)	—	**29 (<1.0)**	**<0.1**
Drowning	7 (<1.0)	—	8 (<1.0)	—	**15 (<1.0)**	—
Other (e.g., Taser, electrocution, or nail gun)	16 (<1.0)	—	10 (<1.0)	—	**26 (<1.0)**	—
Unknown	472 (4.4)	—	141 (5.0)	—	**613 (4.6)**	—
**Location**						
House or apartment	4,177 (39.4)	3.6	1,837 (64.9)	1.5	**6,014 (44.7)**	**2.6**
Street or highway	2,606 (24.6)	2.3	238 (8.4)	0.2	**2,844 (21.2)**	**1.2**
Motor vehicle	1,092 (10.3)	0.9	216 (7.6)	0.2	**1,308 (9.7)**	**0.6**
Parking lot, public garage, or public transport	493 (4.6)	0.4	42 (1.5)	<0.1	**535 (4.0)**	**0.2**
Commercial or retail area	413 (3.9)	0.4	57 (2.0)	<0.1	**470 (3.5)**	**0.2**
Natural area	181 (1.7)	0.2	56 (2.0)	<0.1	**237 (1.8)**	**0.1**
Park, playground, or sports or athletic area	154 (1.5)	0.1	26 (<1.0)	<0.1	**180 (1.3)**	**<0.1**
Bar or nightclub	156 (1.5)	0.1	5 (<1.0)	—	**161 (1.2)**	**<0.1**
Hotel or motel	85 (<1.0)	<0.1	47 (1.7)	<0.1	**132 (<1.0)**	**<0.1**
Jail or prison	89 (<1.0)	<0.1	0 (0)	—	**89 (<1.0)**	**<0.1**
Abandoned house, building, or warehouse	63 (<1.0)	<0.1	16 (<1.0)	—	**79 (<1.0)**	**<0.1**
Supervised residential facility	26 (<1.0)	<0.1	19 (<1.0)	—	**45 (<1.0)**	**<0.1**
Other location^§§^	219 (2.1)	—	58 (2.0)	—	**277 (2.1)**	—
Unknown	856 (8.1)	—	214 (7.6)	—	**1,070 (8.0)**	—
**Relationship of victim to suspect^¶¶^**						
Acquaintance or friend	1,220 (31.6)	1.1	217 (11.7)	0.2	**1,437 (25.1)**	**0.6**
Spouse or intimate partner (current or former)	321 (8.3)	0.3	942 (50.6)	0.8	**1,263 (22.1)**	**0.5**
Other person, known to victim	791 (20.5)	0.7	139 (7.5)	0.1	**930 (16.2)**	**0.4**
Stranger	646 (16.7)	0.6	108 (5.8)	<0.1	**754 (13.2)**	**0.3**
Other relative	291 (7.5)	0.3	148 (7.9)	0.1	**439 (7.7)**	**0.2**
Child***	222 (5.7)	0.2	150 (8.1)	0.1	**372 (6.5)**	**0.2**
Parent***	173 (4.5)	0.2	117 (6.3)	0.1	**290 (5.1)**	**0.1**
Child of suspect's boyfriend or girlfriend (e.g., child killed by mother's boyfriend)	61 (1.6)	<0.1	32 (1.7)	<0.1	**93 (1.6)**	**<0.1**
Rival gang member	71 (1.8)	<0.1	6 (<1.0)	—	**77 (1.3)**	**<0.1**
Other relationship^+++^	67 (1.7)	—	3 (<1.0)	—	**70 (1.2)**	—
**Total**	**10,610 (100.0)**	**9.2**	**2,831 (100.0)**	**2.4**	**13,441 (100.0)**	**5.7**

* Percentages might not total 100% due to rounding.

^†^ Per 100,000 population.

^§^ The following statement can be used as a general guide for interpreting the victim-suspect relationship: “The victim is the [insert relationship] of the suspect.” For example, when a parent kills a child, the relationship is “child,” not “parent” (The victim is the child of the suspect.). Some relationships might not be captured by this sentence (e.g., if the other person is known to the victim or if the victim was a law enforcement officer killed in the line of duty).

^¶^ Data for all violent deaths were collected in 36 states (Alabama, Alaska, Arizona, California, Colorado, Connecticut, Delaware, Georgia, Illinois, Indiana, Iowa, Kansas, Kentucky, Louisiana, Maine, Maryland, Massachusetts, Michigan, Minnesota, Missouri, Nebraska, Nevada, New Hampshire, New Jersey, New Mexico, New York, North Carolina, Ohio, Oklahoma, Oregon, Pennsylvania, Rhode Island, South Carolina, Utah, Vermont, Virginia, Washington, West Virginia, and Wisconsin), and the District of Columbia. Three states (California, Illinois, and Pennsylvania) collected data from a subset of counties in their state. Data for violent deaths that occurred in Illinois include 28 counties that represent 86% of the state’s population (Adams, Boone, Champaign, Cook, DuPage, Effingham, Fulton, Kane, Kankakee, Kendall, Lake, Lasalle, Livingston, Logan, McDonough, McHenry, McLean, Macoupin, Madison, Peoria, Perry, Rock Island, St. Clair, Sangamon, Tazewell, Vermillion, Will, and Winnebago). Data for violent deaths that occurred in Pennsylvania include 39 counties that represent 82.2% of the state’s population (Adams, Allegheny, Armstrong, Beaver, Berks, Blair, Bradford, Bucks, Cambria, Carbon, Centre, Chester, Clarion, Clearfield, Clinton, Columbia, Crawford, Dauphin, Delaware, Fayette, Forest, Greene, Indiana, Jefferson, Lackawanna, Lancaster, Lehigh, Luzerne, Monroe, Montgomery, Montour, Northampton, Philadelphia, Schuylkill, Union, Wayne, Westmoreland, Wyoming, and York). Data for violent deaths that occurred in California include 21 counties that represent 54% of the state’s population (Amador, Butte, Fresno, Humboldt, Imperial, Kern, Kings, Lake, Los Angeles, Marin, Mono, Placer, Sacramento, San Benito, San Diego, San Francisco, San Mateo, Shasta, Siskiyou, Ventura, and Yolo). Denominators for the rates for these three states (California, Illinois, and Pennsylvania) represent only the populations of the counties from which the data were collected.

** Rates are not reported when the number of decedents is <20 or when the characteristic response is “other” or “unknown.”

^††^ Includes persons of any race.

^§§^ Other location includes (in descending order) office building; preschool, school, college, or school bus; synagogue, church, or temple; industrial or construction area; hospital or medical facility; farm; railroad tracks; cemetery, graveyard, or other burial ground; bridge; and other unspecified location.

^¶¶^ Percentage is based on the number of homicide decedents with a known victim-suspect relationship (n = 5,725 [42.6%]; 3,863 [36.4%] males and 1,862 [65.8%] females); victim-to-suspect relationship was unknown for 7,716 decedents.

*** Includes adoptive family members (e.g., adopted child), stepfamily members (e.g., stepparent), and foster family members (e.g., foster child).

^†††^ Other relationship includes (in descending order) the victim was a law enforcement officer injured in the line of duty, and victim was an intimate partner of suspect's parent (e.g., teenager kills mother’s boyfriend).

The highest homicide rate was among adults aged 20–24 years (13.2 per 100,000 population) and was higher among males than among females across all age groups (Table 4). The homicide rate for males aged 20–24 years was 5.3 times the rate for females aged 20–24 years. Among males, the rate of homicide was highest among those aged 20–24 years and 25–29 years (21.8 and 21.3 per 100,000 population, respectively). Among females, the rate of homicide was highest among infants aged <1 year (6.3 per 100,000 population). The overall homicide rate for infants aged <1 year (7.3 per 100,000 population) was 3.3 times the overall rate for children aged 1–4 years (2.2 per 100,000 population) and 14.6 times the rate for children aged 5–9 years (0.5 per 100,000 population).

Non-Hispanic Black males accounted for 58.6% of male homicide victims and 46.3% of all homicides (Table 4). Non-Hispanic Black males had the highest rate of homicide across any racial or ethnic group (40.9 per 100,000 population); this rate was 17.0 times the rate for non-Hispanic Asian or Pacific Islander males (2.4 per 100,000 population), 12.4 times the rate for non-Hispanic White males (3.3 per 100,000 population), 4.8 times the rate for Hispanic males (8.6 per 100,000 population), and 2.2 times the rate for non-Hispanic AI/AN males (18.2 per 100,000 population).

Among females, the homicide rate was highest among non-Hispanic Black persons (6.3 per 100,000 population) (Table 4), followed by non-Hispanic AI/AN persons (5.3 per 100,000 population), Hispanic persons (1.9 per 100,000 population), non-Hispanic White persons (1.7 per 100,000 population), and Asian or Pacific Islander persons (1.0 per 100,000 population).

#### Method, Location of Injury, and Victim-Suspect Relationship

A firearm was used in 71.5% of homicides, followed by sharp instrument (10.6%), blunt instrument (4.2%), personal weapon (e.g., hands, feet, or fists) (3.8%), and hanging, strangulation, or suffocation (2.3%) (Table 4). The method was unknown in 4.6% of homicides. A firearm was the most common method of injury for both males (75.7%) and females (55.4%); however, the firearm homicide rate for males was 5.3 times the rate for females (6.9 versus 1.3 per 100,000 population). A larger proportion of homicides among females than males involved a sharp instrument (15.5% versus 9.3%, respectively); a blunt instrument (7.0% versus 3.5%, respectively); hanging, strangulation, or suffocation (6.6% versus 1.1%, respectively); and a personal weapon (4.9% versus 3.4%, respectively). Among all homicide victims, a house or apartment was the most common location of homicide (44.7%), followed by a street or highway (21.2%), a motor vehicle (9.7%), and a parking lot, public garage, or public transport (4.0%). However, a larger proportion of homicides among females (64.9%) than among males (39.4%) occurred at a house or apartment, whereas a larger proportion of homicides among males (24.6%) than among females (8.4%) occurred on a street or highway.

The relationship of the victim to the suspect was known for 42.6% of homicides (36.4% of males and 65.8% of females) (Table 4). For males, when the relationship was known, the victim-suspect relationship was most often an acquaintance or a friend (31.6%), a person known to the victim but for whom the exact nature of the relationship was unclear (20.5%), a stranger (16.7%), or a current or former intimate partner (8.3%). For females, when the relationship was known, approximately one half (50.6%) were a current or former intimate partner, followed by an acquaintance or a friend (11.7%), a child (8.1%), a parent (6.3%), or a stranger (5.8%).

#### Precipitating Circumstances

Precipitating circumstances were identified in 76.0% of homicides (Table 5). Approximately one in three homicides with known circumstances was precipitated by an argument or conflict (33.7%). Homicides also were commonly precipitated by another crime (26.2%); in 60.0% of those cases, the crime was in progress at the time of the incident. The most frequent types of precipitating crimes were assault or homicide (41.4%), robbery (32.6%), drug trade** (14.7%), burglary (12.9%), motor vehicle theft (4.7%), rape or sexual assault (2.8%), and arson (1.7%) (Supplementary Table S10, https://stacks.cdc.gov/view/cdc/112767). Approximately one sixth (16.9%) of homicides with known circumstances were intimate partner violence related (Table 5). Intimate partner violence–related deaths include deaths related to conflict or violence between current or former intimate partners, and also include deaths associated with intimate partner violence that are not deaths of the intimate partners themselves (e.g., a former boyfriend kills the ex-partner’s new boyfriend). A physical fight between two persons (15.7%) and drug involvement (i.e., drug dealing, drug trade, or drug use) (12.3%) were other common precipitating circumstances.

**TABLE 5:** Number* and percentage^†^ of homicides, by decedent’s sex and precipitating circumstance — National Violent Death Reporting System, 39 states and the district of Columbia, 2018^§^

Precipitating circumstance	Male	Female	Total
	No. (%)	No. (%)	No. (%)
**Mental health or substance use**			
Substance use problem (excludes alcohol)	998 (12.7)	288 (12.3)	**1,286 (12.6)**
Current diagnosed mental health problem	345 (4.4)	182 (7.7)	**527 (5.2)**
Alcohol problem	311 (4.0)	86 (3.7)	**397 (3.9)**
History of ever being treated for a mental health problem	219 (2.8)	119 (5.1)	**338 (3.3)**
Current mental health treatment	115 (1.5)	70 (3.0)	**185 (1.8)**
Current depressed mood	35 (<1.0)	26 (1.1)	**61 (<1.0)**
Other addiction (e.g., gambling or sex)	12 (<1.0)	4 (<1.0)	**16 (<1.0)**
**Interpersonal**			
Intimate partner violence related	674 (8.6)	1,048 (44.6)	**1,722 (16.9)**
Family relationship problem	409 (5.2)	238 (10.1)	**647 (6.3)**
Other relationship problem (nonintimate)	482 (6.1)	107 (4.6)	**589 (5.8)**
Jealousy (lovers’ triangle)	202 (2.6)	108 (4.6)	**310 (3.0)**
Victim of interpersonal violence during past month	97 (1.2)	126 (5.4)	**223 (2.2)**
Perpetrator of interpersonal violence during past month	142 (1.8)	12 (<1.0)	**154 (1.5)**
**Life stressor**			
Argument or conflict	2,752 (35.0)	693 (29.5)	**3,445 (33.7)**
Physical fight (two persons, not a brawl)	1,387 (17.6)	212 (9.0)	**1,599 (15.7)**
Crisis during previous or upcoming 2 weeks	433 (5.5)	222 (9.4)	**655 (6.4)**
History of child abuse or neglect	65 (<1.0)	41 (1.7)	**106 (1.0)**
**Crime and criminal activity**			
Precipitated by another crime	2,195 (27.9)	477 (20.3)	**2,672 (26.2)**
Crime in progress^¶^	1,326 (60.4)	276 (57.9)	**1,602 (60.0)**
Drug involvement	1,122 (14.3)	129 (5.5)	**1,251 (12.3)**
Gang-related	990 (12.6)	95 (4.0)	**1,085 (10.6)**
**Homicide circumstance**			
Drive-by shooting	791 (10.1)	98 (4.2)	**889 (8.7)**
Walk-by assault	600 (7.6)	66 (2.8)	**666 (6.5)**
Victim used a weapon	612 (7.8)	29 (1.2)	**641 (6.3)**
Caretaker abuse or neglect led to death	261 (3.3)	198 (8.4)	**459 (4.5)**
Mentally ill suspect**	163 (2.1)	156 (6.6)	**319 (3.1)**
Justifiable self defense	283 (3.6)	7 (<1.0)	**290 (2.8)**
Random violence	169 (2.1)	63 (2.7)	**232 (2.3)**
Victim was a bystander	134 (1.7)	88 (3.7)	**222 (2.2)**
Brawl	191 (2.4)	10 (<1.0)	**201 (2.0)**
Victim was an intervener assisting a crime victim	100 (1.3)	21 (<1.0)	**121 (1.2)**
Prostitution	29 (<1.0)	28 (1.2)	**57 (<1.0)**
Stalking	20 (<1.0)	34 (1.4)	**54 (<1.0)**
Victim was a police officer on duty	39 (<1.0)	4 (<1.0)	**43 (<1.0)**
Mercy killing	5 (<1.0)	16 (<1.0)	**21 (<1.0)**
Hate crime	14 (<1.0)	7 (<1.0)	**21 (<1.0)**
**Total^††^**	**7,861 (74.1)**	**2,350 (83.0)**	**10,211 (76.0)**

* Includes homicides with one or more precipitating circumstances. Total numbers do not equal the sums of the columns because more than one circumstance could have been present per decedent.

^†^ Denominator includes those homicides with one or more precipitating circumstances. The sums of percentages in columns exceed 100% because more than one circumstance could have been present per decedent.

^§^ Data for all violent deaths were collected in 36 states (Alabama, Alaska, Arizona, California, Colorado, Connecticut, Delaware, Georgia, Illinois, Indiana, Iowa, Kansas, Kentucky, Louisiana, Maine, Maryland, Massachusetts, Michigan, Minnesota, Missouri, Nebraska, Nevada, New Hampshire, New Jersey, New Mexico, New York, North Carolina, Ohio, Oklahoma, Oregon, Pennsylvania, Rhode Island, South Carolina, Utah, Vermont, Virginia, Washington, West Virginia, and Wisconsin), and the District of Columbia. Three states (California, Illinois, and Pennsylvania) collected data from a subset of counties in their state. Data for violent deaths that occurred in Illinois include 28 counties that represent 86% of the state’s population (Adams, Boone, Champaign, Cook, DuPage, Effingham, Fulton, Kane, Kankakee, Kendall, Lake, Lasalle, Livingston, Logan, McDonough, McHenry, McLean, Macoupin, Madison, Peoria, Perry, Rock Island, St. Clair, Sangamon, Tazewell, Vermillion, Will, and Winnebago). Data for violent deaths that occurred in Pennsylvania include 39 counties that represent 82.2% of the state’s population (Adams, Allegheny, Armstrong, Beaver, Berks, Blair, Bradford, Bucks, Cambria, Carbon, Centre, Chester, Clarion, Clearfield, Clinton, Columbia, Crawford, Dauphin, Delaware, Fayette, Forest, Greene, Indiana, Jefferson, Lackawanna, Lancaster, Lehigh, Luzerne, Monroe, Montgomery, Montour, Northampton, Philadelphia, Schuylkill, Union, Wayne, Westmoreland, Wyoming, and York). Data for violent deaths that occurred in California include 21 counties that represent 54% of the state’s population (Amador, Butte, Fresno, Humboldt, Imperial, Kern, Kings, Lake, Los Angeles, Marin, Mono, Placer, Sacramento, San Benito, San Diego, San Francisco, San Mateo, Shasta, Siskiyou, Ventura, and Yolo). Denominators for the rates for these three states (California, Illinois, and Pennsylvania) represent only the populations of the counties from which the data were collected.

^¶^ Denominator includes those decedents involved in an incident that was precipitated by another crime.

** Mentally ill suspect is endorsed for deaths in which the suspect’s attack on decedent was believed to be the direct result of a mental health problem (e.g., schizophrenia or other psychotic condition, depression, or posttraumatic stress disorder).

^††^ Circumstances were unknown for 3,230 decedents (2,749 males and 481 females); total number of homicide decedents = 13,441 (10,610 males and 2,831 females).

Among the identified homicide circumstances, several differences were noted by decedent’s sex, and intimate partner violence accounted for the largest percentage difference (Table 5). Intimate partner violence was a precipitating circumstance for approximately 44.6% of homicides among females but only 8.6% of homicides among males. A larger proportion of female victims (8.4%) than male victims (3.3%) of homicide also resulted from caretaker abuse or neglect. A larger proportion of homicides of female victims were perpetrated by a suspect with a mental health problem (e.g., schizophrenia or other psychotic conditions, depression, or PTSD; 6.6%) than homicides of male victims (2.1%). A larger proportion of homicides of males than females were preceded by a physical fight (17.6% versus 9.0%, respectively), involved drugs (14.3% versus 5.5%, respectively), and were gang related (12.6% versus 4.0%, respectively). A larger proportion of male victims (7.8%) than female victims (1.2%) also was reported to have used a weapon during the incident.

#### Homicide Suspects

In addition to summarizing NVDRS victim data, this report highlights information about suspected perpetrators (suspects) of homicides for cases with suspect information. Suspect information can be used to support violence prevention efforts by improving understanding of the contextual factors related to fatal violence perpetration and of the circumstances surrounding these incidents. In NVDRS, a suspect is defined as a person believed by law enforcement to have perpetrated a fatal injury in an incident (*6*). For 2018, a total of 39 states and the District of Columbia collected data on 8,051 homicide incidents with an identified suspect, representing 63.4% of all homicide incidents. Information was collected on 9,931 suspects, as certain incidents had multiple suspects. Suspect age was missing for 26.2% (n = 2,600) of suspects. This report includes analyses for 7,331 suspects in the 6,071 homicide incidents (47.8% of all homicide incidents) in which a suspect was identified and the suspect’s age was known (Table 6).

**TABLE 6:** Number and percentage* of homicides, by selected demographic characteristics of suspect, victim-suspect relationship, suspect’s mental health or substance use, and homicide circumstance — National Violent Death Reporting System, 39 states and the District of Columbia, 2018^†^

Characteristic	Suspect age group (yrs)					Total
	<18	18–24	25–44	45–64	≥65	
	No. (%)^§^	No. (%)^§^	No. (%)^§^	No. (%)^§^	No. (%)^§^	No. (%)^§^
**Sex**						
Male	426 (91.0)	1,880 (88.8)	3,020 (86.5)	881 (85.0)	197 (90.4)	**6,404 (87.4)**
Female	37 (7.9)	226 (10.7)	461 (13.2)	154 (14.9)	21 (9.6)	**899 (12.3)**
Unknown	5 (1.1)	12 (<1.0)	9 (<1.0)	2 (<1.0)	0 (0)	**28 (<1.0)**
**Race/Ethnicity**						
Black, non-Hispanic	290 (62.0)	1,303 (61.5)	1,798 (51.5)	373 (36.0)	35 (16.1)	**3,799 (51.8) **
White, non-Hispanic	87 (18.6)	407 (19.2)	1,021 (29.3)	518 (50.0)	149 (68.3)	**2,182 (29.8)**
Hispanic^¶^	37 (7.9)	189 (8.9)	322 (9.2)	63 (6.1)	9 (4.1)	**620 (8.5)**
Asian or Pacific Islander, non-Hispanic	0 (0.)	15 (<1.0)	35 (1.0)	18 (1.7)	5 (2.3)	**73 (<1.0)**
American Indian or Alaska Native, non-Hispanic	4 (<1.0)	12 (<1.0)	48 (1.4)	6 (<1.0)	2 (<1.0)	**72 (<1.0)**
Unknown	50 (10.7)	192 (9.1)	266 (7.6)	59 (5.7)	18 (8.3)	**585 (8.0)**
Relationship of victim to suspect**						
Acquaintance or friend	98 (31.2)	447 (34.0)	666 (27.0)	192 (22.2)	21 (10.4)	**1,424 (27.6)**
Spouse or intimate partner (current or former)	8 (2.5)	124 (9.4)	521 (21.2)	333 (38.5)	114 (56.4)	**1,100 (21.3)**
Other person, known to victim	46 (14.6)	230 (17.5)	401 (16.3)	109 (12.6)	15 (7.4)	**801 (15.5)**
Stranger	77 (24.5)	232 (17.6)	333 (13.5)	65 (7.5)	10 (5.0)	**717 (13.9)**
Other relative^††^	35 (11.1)	76 (5.8)	161 (6.5)	66 (7.6)	17 (8.4)	**355 (6.9)**
Child^§§^	10 (3.2)	82 (6.2)	167 (6.8)	45 (5.2)	11 (5.4)	**315 (6.1)**
Parent^§§^	24 (7.6)	48 (3.6)	107 (4.3)	48 (5.6)	8 (4.0)	**235 (4.6)**
Child of suspect's boyfriend or girlfriend (e.g., child killed by mother's boyfriend)	1 (<1.0)	24 (1.8)	56 (2.3)	4 (<1.0)	2 (<1.0)	**87 (1.7)**
Rival gang member	8 (2.5)	31 (2.4)	26 (1.1)	0 (0)	0 (0)	**65 (1.3)**
Intimate partner of suspect's parent (e.g., teenager kills mother’s boyfriend)	7 (2.2)	13 (<1.0)	9 (<1.0)	1 (<1.0)	1 (<1.0)	**31 (<1.0)**
Victim was a law enforcement officer on duty	0 (0)	9 (<1.0)	16 (<1.0)	1 (<1.0)	3 (1.5)	**29 (<1.0)**
**Mental health or substance use^¶¶^**						
Suspected other substance use by suspect	27 (7.8)	119 (7.5)	297 (10.1)	76 (7.8)	4 (1.9)	**523 (8.6)**
Suspected alcohol use by suspect	14 (4.1)	77 (4.9)	239 (8.1)	112 (11.5)	11 (5.2)	**453 (7.5)**
Mentally ill suspect***	9 (2.6)	47 (3.0)	144 (4.9)	69 (7.1)	21 (9.9)	**290 (4.8)**
Suspect had a developmental disability	1 (<1.0)	3 (<1.0)	5 (<1.0)	4 (<1.0)	1 (<1.0)	**14 (<1.0)**
**Other circumstance of suspect^†††^**						
Prior contact with law enforcement	38 (11.0)	174 (11.0)	386 (13.1)	99 (10.1)	10 (4.7)	**707 (11.6)**
Suspect attempted suicide after incident^§§§^	6 (1.7)	30 (1.9)	192 (6.5)	171 (17.5)	82 (38.5)	**481 (7.9)**
Suspect recently released from an institution	3 (<1.0)	38 (2.4)	69 (2.3)	21 (2.1)	4 (1.9)	**135 (2.2)**
**Homicide circumstance**						
Precipitated by another crime	114 (38.9)	463 (32.8)	733 (27.5)	202 (22.0)	30 (14.9)	**1,542 (28.1)**
Intimate partner violence related	25 (8.5)	171 (12.1)	680 (25.5)	388 (42.3)	117 (58.2)	**1,381 (25.1)**
Drug involvement	58 (19.8)	228 (16.1)	340 (12.7)	53 (5.8)	4 (2.0)	**683 (12.4)**
Victim used a weapon	27 (9.2)	122 (8.6)	202 (7.6)	57 (6.2)	10 (5.0)	**418 (7.6)**
Gang related	29 (9.9)	157 (11.1)	193 (7.2)	9 (<1.0)	0 (0)	**388 (7.1)**
Drive by shooting	20 (6.8)	114 (8.1)	126 (4.7)	9 (<1.0)	0 (0)	**269 (4.9)**
Jealousy (lovers’ triangle)	5 (1.7)	47 (3.3)	139 (5.2)	54 (5.9)	4 (2.0)	**249 (4.5)**
Brawl (mutual physical fight)	10 (3.4)	40 (2.8)	58 (2.2)	6 (<1.0)	0 (0)	**114 (2.1)**
Random violence	8 (2.7)	33 (2.3)	54 (2.0)	11 (1.2)	2 (<1.0)	**108 (2.0)**
Victim was a bystander	10 (3.4)	38 (2.7)	40 (1.5)	6 (<1.0)	0 (0)	**94 (1.7)**
Stalking	0 (0)	6 (<1.0)	21 (<1.0)	12 (1.3)	3 (1.5)	**42 (<1.0)**
Prostitution	1 (<1.0)	9 (<1.0)	24 (<1.0)	4 (<1.0)	0 (0)	**38 (<1.0)**
Hate crime	0 (0)	3 (<1.0)	2 (<1.0)	2 (<1.0)	0 (0)	**7 (<1.0)**
**Total**	**468**	**2,118**	**3,490**	**1,037**	**218**	**7,331**

* Percentages might not total 100% due to rounding. There were 12,693 homicide incidents overall and 9,331 suspects from 8,051 incidents with suspect information. Of the total number of homicide incidents, 6,071 (47.8%) had known suspect age, resulting in 7,331 suspects (age was unknown for n = 2,600 (26.2%) of suspects). Some incidents had >1 suspect. Denominators for suspect characteristics and circumstances vary by the availability of known information and are specified in separate footnotes.

^†^ Data for all violent deaths were collected in 36 states (Alabama, Alaska, Arizona, California, Colorado, Connecticut, Delaware, Georgia, Illinois, Indiana, Iowa, Kansas, Kentucky, Louisiana, Maine, Maryland, Massachusetts, Michigan, Minnesota, Missouri, Nebraska, Nevada, New Hampshire, New Jersey, New Mexico, New York, North Carolina, Ohio, Oklahoma, Oregon, Pennsylvania, Rhode Island, South Carolina, Utah, Vermont, Virginia, Washington, West Virginia, and Wisconsin), and the District of Columbia. Three states (California, Illinois, and Pennsylvania) collected data from a subset of counties in their state. Data for violent deaths that occurred in Illinois include 28 counties that represent 86% of the state’s population (Adams, Boone, Champaign, Cook, DuPage, Effingham, Fulton, Kane, Kankakee, Kendall, Lake, Lasalle, Livingston, Logan, McDonough, McHenry, McLean, Macoupin, Madison, Peoria, Perry, Rock Island, St. Clair, Sangamon, Tazewell, Vermillion, Will, and Winnebago). Data for violent deaths that occurred in Pennsylvania include 39 counties that represent 82.2% of the state’s population (Adams, Allegheny, Armstrong, Beaver, Berks, Blair, Bradford, Bucks, Cambria, Carbon, Centre, Chester, Clarion, Clearfield, Clinton, Columbia, Crawford, Dauphin, Delaware, Fayette, Forest, Greene, Indiana, Jefferson, Lackawanna, Lancaster, Lehigh, Luzerne, Monroe, Montgomery, Montour, Northampton, Philadelphia, Schuylkill, Union, Wayne, Westmoreland, Wyoming, and York). Data for violent deaths that occurred in California include 21 counties that represent 54% of the state’s population (Amador, Butte, Fresno, Humboldt, Imperial, Kern, Kings, Lake, Los Angeles, Marin, Mono, Placer, Sacramento, San Benito, San Diego, San Francisco, San Mateo, Shasta, Siskiyou, Ventura, and Yolo). Denominators for the rates for these three states (California, Illinois, and Pennsylvania) represent only the populations of the counties from which the data were collected.

^§^Percentage is based on the total number of suspects within each age group (i.e., column totals at the bottom of the table).

^¶^ Includes persons of any race.

** Percentage is based on the number of homicide suspects with a known age and victim-suspect relationship (n = 5,159; aged <18 years = 314; aged 18–24 years = 1,316; aged 25–44 years = 2,463; aged 45–64 years = 864; aged ≥65 years = 202); victim-suspect relationship was unknown for 2,172 suspects. The victim-suspect relationship should be interpreted using the following statement: “The victim is the [insert relationship] of the suspect,” with the exception of the caregiver relationship.

^††^ Other relative includes other family member (e.g., cousin or uncle), sibling, grandparent, in-law, or grandchild.

^§§^ Includes adoptive family members (e.g., adopted child), stepfamily members (e.g., stepparent), and foster family members (e.g., foster child).

^¶¶^ Percentage is based on the number of homicide incidents (n = 6,071; aged <18 years = 344; aged 18–24 years = 1,586; aged 25–44 years = 2,951; aged 45–64 years = 977; and aged ≥65 years = 213) with the count representing the total number of suspects having that characteristic.

*** Mentally ill suspect is endorsed for deaths in which the suspect’s attack on decedent was believed to be the direct result of a mental health problem (e.g., schizophrenia or other psychotic condition, depression, or posttraumatic stress disorder).

^†††^ Percentage is based on the number of homicide incidents with known suspect age and decedent circumstances (n = 5,494; primary suspect: aged <18 years = 293; aged 18–24 years = 1,412; aged 25–44 years = 2,670; aged 45–64 years = 918; and aged ≥65 years = 201). The characteristic applies to one or more decedents in the incident.

^§§§^ Number and percentage of suspect suicide attempts that were fatal, based on the number who attempted suicide: n = 389 (80.9%); aged <18 years = 5 (83.3%); aged 18–24 years = 18 (60.0%); aged 25–44 years = 151 (78.6%); aged 45–64 years = 138 (80.7%); and aged ≥65 years = 77 (93.9%).

**Suspect demographics**. Overall, 87.4% of suspects in homicides were male and 12.3% were female (Table 6). For <1% of suspects, the sex was unknown. Regarding suspect race and ethnicity, 51.8% of suspects were non-Hispanic Black, followed by non-Hispanic White (29.8%) and Hispanic (8.5%). Non-Hispanic AI/AN and Asian or Pacific Islander persons each represented <1.0% of homicide suspects. Race and ethnicity were unknown for 8.0% of suspects.

**Victim-suspect relationship.** In cases in which the relationship of the victim to the suspect was known, 27.6% of suspects were accused of fatally injuring an acquaintance or friend, and 21.3% were suspected of fatally injuring a current or former spouse or intimate partner (Table 6). Approximately one sixth (15.5%) were suspected of fatally injuring someone else whom they knew but who did not fall into the categories of a friend, family member, rival gang member, or current or former intimate partner. In 13.9% of homicides, the victim was a stranger to the suspect. In addition, 6.9% and 6.1% of victims in homicides were another relative or a child of the suspect, respectively.

**Suspect substance use, mental health, and law enforcement contact**. Approximately one in 12 (8.6%) suspects were perceived as being under the influence of substances (other than alcohol), and 7.5% were suspected of using alcohol in the hours preceding the fatal incident (Table 6). Approximately one in 20 (4.8%) of suspects were thought to have a mental health problem (e.g., schizophrenia or other psychotic conditions, depression, or PTSD) that directly contributed to the fatal injury, and <1.0% had a developmental disability. Approximately one in 10 suspects (11.6%) had prior contact with law enforcement, 7.9% attempted suicide after the homicide incident, and 2.2% were reported as having been recently released from an institution (e.g., jail, hospital, or psychiatric hospital).

**Most common homicide circumstances**. Approximately one third (28.1%) of suspects were involved in incidents that were precipitated by another crime (Table 6). One fourth of suspects (25.1%) were involved in homicides for which intimate partner violence was a precipitating circumstance, and 12.4% were part of incidents with drug involvement.

#### Most Common Demographic Characteristics, Victim-Suspect Relationships, Suspect Circumstances, and Homicide Circumstances by Suspect Age Group

##### Suspects Aged <18 Years

Among suspects aged <18 years, 91.0% were male, and 7.9% were female (Table 6). The majority of suspects in this age group were non-Hispanic Black persons (62.0%), followed by non-Hispanic White (18.6%), Hispanic (7.9%), non-Hispanic Asian or Pacific Islander persons (<1.0%), and AI/AN (<1.0%) persons. Suspects within this age group were primarily suspected of fatally injuring an acquaintance or friend (31.2%) or a stranger (24.5%). Approximately one in 11 (7.8%) suspects were perceived to be under the influence of a substance other than alcohol at the time of the incident, and 11.0% had prior contact with law enforcement. The most common precipitating circumstances for suspects in this age group were that the fatal injury was precipitated by another crime (38.9%), was drug involved (19.8%), or was gang related (9.9%).

##### Suspects Aged 18–24 Years

Among suspects aged 18–24 years, 88.8% were male, and 10.7% were female (Table 6). More than one half (61.5%) of suspects in this age group were non-Hispanic Black persons, and 19.2% were non-Hispanic White persons. Suspects aged 18–24 years were primarily suspected of fatally injuring an acquaintance or friend (34.0%), a stranger (17.6%), or another person whom they knew but who did not fall into the categories of friend, family member, rival gang member, or current or former intimate partner (17.5%). Approximately one in 13 suspects (7.5%) were under the influence of a substance other than alcohol at the time of the incident, and 11.0% had prior contact with law enforcement. The most common precipitating circumstances for suspects in this age group were that the fatal injury was precipitated by another crime (32.8%), involved drugs (16.1%), or was intimate partner violence related (12.1%).

##### Suspects Aged 25–44 Years

Among suspects aged 25–44 years, 86.5% were male, and 13.2% were female (Table 6). Approximately one half (51.5%) of suspects in this age group were non-Hispanic Black persons, and 29.3% were non-Hispanic White persons. Suspects aged 25–44 years were primarily suspected of fatally injuring an acquaintance or friend (27.0%) or a current or former spouse or an intimate partner (21.2%). Approximately one in 10 suspects (10.1%) was perceived to be under the influence of a substance other than alcohol at the time of the incident, and 13.1% had prior contact with law enforcement. The most common incident circumstances for suspects in this age group were that the fatal injury was precipitated by another crime (27.5%), was intimate partner violence related (25.5%), or was drug involved (12.7%).

##### Suspects Aged 45–64 Years

Among suspects aged 45–64 years, 85.0% were male, and 14.9% were female (Table 6). One half of suspects in this age group were non-Hispanic White (50.0%) persons, and 36.0% were non-Hispanic Black persons. Suspects aged 45–64 years were primarily suspected of fatally injuring a current or former spouse or intimate partner (38.5%) or an acquaintance or a friend (22.2%). Approximately one in 10 (11.5%) suspects was suspected of using alcohol in the hours preceding the incident, and 17.5% attempted suicide after the incident. The most common incident circumstances for suspects in this age group were that the fatal injury was intimate partner violence–related (42.3%), the injury was precipitated by another crime (22.0%), or the victim used a weapon during the fatal incident (6.2%).

##### Suspects Aged ≥65 Years

Among suspects aged ≥65 years, 90.4% were male, and 9.6% were female (Table 6). Over two thirds of suspects in this age group were non-Hispanic White (68.3%) persons, and 16.1% were non-Hispanic Black persons. Suspects aged ≥65 years were primarily suspected of fatally injuring a current or former spouse or intimate partner (56.4%) or an acquaintance or a friend (10.4%). Approximately one in 10 (9.9%) suspects were believed to have a mental health problem (e.g., schizophrenia or other psychotic conditions, depression, or PTSD) that directly influenced the fatal injury, and 38.5% attempted suicide after the incident. The most common circumstances for suspects in this age group were that the fatal injury was intimate partner violence-related (58.2%), and the incident was precipitated by another crime (14.9%).

### Legal Intervention Deaths

#### Sex, Age Group, and Race and Ethnicity

For 2018, a total of 39 states and the District of Columbia collected data on 757 legal intervention death incidents involving 764 deaths. Almost all legal intervention deaths were among males (95.2%) (Table 7). The highest rate of legal intervention death by age group was among males aged 30–34 years (1.5 per 100,000 population), followed by males aged 35–44 years and 25–29 years both at 1.3 per 100,000 population. Although non-Hispanic White males accounted for nearly half (46.3%) of all legal intervention deaths, non-Hispanic AI/AN males had the highest legal intervention death rate (2.6 per 100,000 population), representing a rate 5.2 times the rate for non-Hispanic White males (0.5 per 100,000 population). The legal intervention death rate for non-Hispanic Black males (1.3 per 100,000 population) was 2.6 times the rate for non-Hispanic White males. The legal intervention death rate for Hispanic males was 0.8 per 100,000 population.

**TABLE 7:** Number, percentage,* and rate^†^ of legal intervention^§^ deaths, by selected demographic characteristics of decedent, method used, and location in which injury occurred — National Violent Death Reporting System, 39 states and the District of Columbia, 2018^¶^

Characteristic	Male		Female		Total	
	No. (%)	Rate	No. (%)	Rate	No. (%)	Rate
**Age group (yrs)**						
<10	0 (0)	—**	0 (0)	—	**0 (0)**	**—**
10–14	1 (<1.0)	—	0 (0)	—	**1 (<1.0)**	**—**
15–19	39 (5.4)	0.5	1 (2.7)	—	**40 (5.2)**	**0.3**
20–24	84 (11.6)	1.0	2 (5.4)	—	**86 (11.3)**	**0.5**
25–29	113 (15.5)	1.3	5 (13.5)	—	**118 (15.4)**	**0.7**
30–34	118 (16.2)	1.5	7 (18.9)	—	**125 (16.4)**	**0.8**
35–44	190 (26.1)	1.3	10 (27.0)	—	**200 (26.2)**	**0.7**
45–54	96 (13.2)	0.7	7 (18.9)	—	**103 (13.5)**	**0.3**
55–64	58 (8.0)	0.4	3 (8.1)	—	**61 (8.0)**	**0.2**
65–74	22 (3.0)	0.2	2 (5.4)	—	**24 (3.1)**	**0.1**
75–84	6 (<1.0)	—	0 (0)	—	**6 (<1.0)**	**—**
≥85	0 (0)	—	0 (0)	—	**0 (0)**	**—**
**Race/Ethnicity**						
White, non-Hispanic	354 (48.7)	0.5	21 (56.8)	<0.1	**375 (49.1)**	**0.3**
Black, non-Hispanic	190 (26.1)	1.3	9 (24.3)	—	**199 (26.0)**	**0.6**
American Indian or Alaska Native, non-Hispanic	28 (3.9)	2.6	1 (2.7)	—	**29 (3.8)**	**1.3**
Asian or Pacific Islander, non-Hispanic	13 (1.8)	—	0 (0)	—	**13 (1.7)**	**—**
Hispanic^††^	138 (19.0)	0.8	6 (16.2)	—	**144 (18.8)**	**0.4**
Other race or ethnicity	4 (<1.0)	—	0 (0)	—	**4 (<1.0)**	**—**
**Method**						
Firearm	659 (90.6)	0.6	29 (78.4)	<0.1	**688 (90.1)**	**0.3**
Motor vehicles (e.g., buses, motorcycles, other transport vehicles)	22 (3.0)	<0.1	6 (16.2)	—	**28 (3.7)**	**<0.1**
Personal weapons (e.g., hands, feet, or fists)	8 (1.1)	—	0 (0)	—	**8 (1.0)**	**—**
Poisoning	6 (<1.0)	—	0 (0)	—	**6 (<1.0)**	**—**
Hanging, strangulation, or suffocation	6 (<1.0)	—	0 (0)	—	**6 (<1.0)**	**—**
Blunt instrument	3 (<1.0)	—	0 (0)	—	**3 (<1.0)**	**—**
Drowning	2 (<1.0)	—	0 (0)	—	**2 (<1.0)**	**—**
Other (e.g., Taser, electrocution, or nail gun)	12 (1.7)	—	0 (0)	—	**12 (1.6)**	**—**
Unknown	9 (1.2)	—	2 (5.4)	—	**11 (1.4)**	**—**
**Location of injury**						
House or apartment	259 (35.6)	0.2	13 (35.1)	—	**272 (35.6)**	**0.1**
Street or highway	188 (25.9)	0.2	8 (21.6)	—	**196 (25.7)**	**<0.1**
Motor vehicle	73 (10.0)	<0.1	8 (21.6)	—	**81 (10.6)**	**<0.1**
Parking lot, public garage, or public transport	47 (6.5)	<0.1	3 (8.1)	—	**50 (6.5)**	**<0.1**
Commercial or retail area	36 (5.0)	<0.1	1 (2.7)	—	**37 (4.8)**	**<0.1**
Natural area	22 (3.0)	<0.1	0 (0)	—	**22 (2.9)**	**<0.1**
Hotel or motel	16 (2.2)	—	0 (0)	—	**16 (2.1)**	**—**
Park, playground, or sports or athletic area	10 (1.4)	—	0 (0)	—	**10 (1.3)**	**—**
Jail or prison	10 (1.4)	—	0 (0)	—	**10 (1.3)**	**—**
Other location^§§^	44 (6.1)	—	1 (2.7)	—	**45 (5.9)**	**—**
Unknown	22 (3.0)	—	3 (8.1)	—	**25 (3.3)**	**—**
**Total**	**727 (100.0)**	**0.6**	**37 (100.0)**	**<0.1**	**764 (100.0)**	**0.3**

* Percentages might not total 100% due to rounding.

^†^ Per 100,000 population.

^§^ The term legal intervention does not denote the lawfulness or legality of the circumstances surrounding the death.

^¶^ Data for all violent deaths were collected in 36 states (Alabama, Alaska, Arizona, California, Colorado, Connecticut, Delaware, Georgia, Illinois, Indiana, Iowa, Kansas, Kentucky, Louisiana, Maine, Maryland, Massachusetts, Michigan, Minnesota, Missouri, Nebraska, Nevada, New Hampshire, New Jersey, New Mexico, New York, North Carolina, Ohio, Oklahoma, Oregon, Pennsylvania, Rhode Island, South Carolina, Utah, Vermont, Virginia, Washington, West Virginia, and Wisconsin), and the District of Columbia. Three states (California, Illinois, and Pennsylvania) collected data from a subset of counties in their state. Data for violent deaths that occurred in Illinois include 28 counties that represent 86% of the state’s population (Adams, Boone, Champaign, Cook, DuPage, Effingham, Fulton, Kane, Kankakee, Kendall, Lake, Lasalle, Livingston, Logan, McDonough, McHenry, McLean, Macoupin, Madison, Peoria, Perry, Rock Island, St. Clair, Sangamon, Tazewell, Vermillion, Will, and Winnebago). Data for violent deaths that occurred in Pennsylvania include 39 counties that represent 82.2% of the state’s population (Adams, Allegheny, Armstrong, Beaver, Berks, Blair, Bradford, Bucks, Cambria, Carbon, Centre, Chester, Clarion, Clearfield, Clinton, Columbia, Crawford, Dauphin, Delaware, Fayette, Forest, Greene, Indiana, Jefferson, Lackawanna, Lancaster, Lehigh, Luzerne, Monroe, Montgomery, Montour, Northampton, Philadelphia, Schuylkill, Union, Wayne, Westmoreland, Wyoming, and York). Data for violent deaths that occurred in California include 21 counties that represent 54% of the state’s population (Amador, Butte, Fresno, Humboldt, Imperial, Kern, Kings, Lake, Los Angeles, Marin, Mono, Placer, Sacramento, San Benito, San Diego, San Francisco, San Mateo, Shasta, Siskiyou, Ventura, and Yolo). Denominators for the rates for these three states (California, Illinois, and Pennsylvania) represent only the populations of the counties from which the data were collected.

** Rates are not reported when number of decedents is <20 or when characteristic response is “other” or “unknown.”

^††^ Includes persons of any race.

^§§^ Other location includes (in descending order) office building; bar or nightclub; hospital or medical facility; farm; preschool, school, college, or school bus; abandoned house, building, or warehouse; synagogue, church, or temple; industrial or construction area; supervised residential facility; bridge; and other unspecified location.

#### Method and Location of Injury

A firearm was used in most (90.1%) legal intervention deaths (Table 7). Legal intervention deaths occurred most frequently in a house or apartment (35.6%), followed by a street or highway (25.7%) or a motor vehicle (10.6%).

#### Precipitating Circumstances

Precipitating circumstances were identified in 96.1% of legal intervention deaths (Table 8). When a specific type of crime was known to have precipitated a legal intervention death (n = 633), the type of crime was most frequently assault or homicide (61.8%), followed by other crime (30.5%), motor vehicle theft (9.3%), robbery (8.5%), burglary (7.0%), and drug trade (1.9%) (Supplementary Table S13, https://stacks.cdc.gov/view/cdc/112767). Among the legal intervention deaths that were precipitated by another crime (86.2%), a crime was reportedly in progress at the time of the incident in approximately three fourths (72.2%) of the deaths (Table 8). The decedent reportedly used a weapon in 73.4% of cases. In 27.7% of legal intervention deaths with known circumstances, a substance use problem (other than alcohol) was reported as a contributing factor, and 19.2% of decedents reportedly had a current diagnosed mental health problem. An argument or conflict precipitated 15.8% of legal intervention deaths, and a recent or impending crisis during the previous or upcoming 2 weeks was reported in 12.0% of legal intervention deaths. Among legal intervention deaths with known circumstances, intimate partner violence (10.4%), being a perpetrator of interpersonal violence during the past month (8.6%), family relationship problem (8.4%) and drug involvement (5.4%) were other notable precipitating circumstances.

**TABLE 8:** Number* and percentage^†^ of legal intervention^§^ deaths, by decedent’s sex and precipitating circumstance — National Violent Death Reporting System, 39 states and the District of Columbia, 2018^¶^

Precipitating circumstance	Male	Female	Total
	No. (%)	No. (%)	No. (%)
**Mental health or substance use**			
Substance use problem (excludes alcohol)	194 (27.6)	9 (28.1)	**203 (27.7)**
Current diagnosed mental health problem	133 (18.9)	8 (25.0)	**141 (19.2)**
History of ever being treated for a mental health problem	81 (11.5)	5 (15.6)	**86 (11.7)**
Alcohol problem	74 (10.5)	2 (6.3)	**76 (10.4)**
Current mental health treatment	42 (6.0)	3 (9.4)	**45 (6.1)**
Current depressed mood	36 (5.1)	0 (0)	**36 (4.9)**
Other addiction (e.g., gambling or sex)	3 (<1.0)	0 (0)	**3 (<1.0)**
**Interpersonal**			
Intimate partner violence-related	75 (10.7)	1 (3.1)	**76 (10.4)**
Perpetrator of interpersonal violence during past month	63 (9.0)	0 (0)	**63 (8.6)**
Family relationship problem	62 (8.8)	0 (0)	**62 (8.4)**
Other relationship problem (nonintimate)	23 (3.3)	0 (0)	**23 (3.1)**
Jealousy (lovers’ triangle)	7 (<1.0)	0 (0)	**7 (<1.0)**
Victim of interpersonal violence during past month	2 (<1.0)	0 (0)	**2 (<1.0)**
**Life stressor**			
Argument or conflict	112 (16.0)	4 (12.5)	**116 (15.8)**
Crisis during previous or upcoming 2 weeks	87 (12.4)	1 (3.1)	**88 (12.0)**
Physical fight (two persons, not a brawl)	64 (9.1)	1 (3.1)	**65 (8.9)**
History of child abuse or neglect	4 (<1.0)	0 (0)	**4 (<1.0)**
**Crime and criminal activity**			
Precipitated by another crime	606 (86.3)	27 (84.4)	**633 (86.2)**
Crime in progress**	438 (72.3)	19 (70.4)	**457 (72.2)**
Drug involvement	37 (5.3)	3 (9.4)	**40 (5.4)**
Gang related	12 (1.7)	0 (0)	**12 (1.6)**
**Legal intervention**			
Victim used a weapon	518 (73.8)	21 (65.6)	**539 (73.4)**
Brawl	12 (1.7)	0 (0)	**12 (1.6)**
Victim was a bystander	1 (<1.0)	3 (9.4)	**4 (<1.0)**
Random violence	3 (<1.0)	0 (0)	**3 (<1.0)**
Stalking	3 (<1.0)	0 (0)	**3 (<1.0)**
Victim was an intervener assisting a crime victim	2 (<1.0)	0 (0)	**2 (<1.0)**
Caretaker abuse or neglect led to death	1 (<1.0)	0 (0)	**1 (<1.0)**
Prostitution	0 (0)	1 (3.1)	**1 (<1.0)**
**Total^††^**	**702 (96.6)**	**32 (86.5)**	**734 (96.1)**

* Includes deaths with one or more precipitating circumstances. Total numbers do not equal the sums of the columns because more than one circumstance could have been present per decedent.

^†^ Denominator includes those deaths with one or more precipitating circumstances. The sums of percentages in columns exceed 100% because more than one circumstance could have been present per decedent.

^§^ The term legal intervention does not denote the lawfulness or legality of the circumstances surrounding the death.

^¶^ Data for all violent deaths were collected in 36 states (Alabama, Alaska, Arizona, California, Colorado, Connecticut, Delaware, Georgia, Illinois, Indiana, Iowa, Kansas, Kentucky, Louisiana, Maine, Maryland, Massachusetts, Michigan, Minnesota, Missouri, Nebraska, Nevada, New Hampshire, New Jersey, New Mexico, New York, North Carolina, Ohio, Oklahoma, Oregon, Pennsylvania, Rhode Island, South Carolina, Utah, Vermont, Virginia, Washington, West Virginia, and Wisconsin), and the District of Columbia. Three states (California, Illinois, and Pennsylvania) collected data from a subset of counties in their state. Data for violent deaths that occurred in Illinois include 28 counties that represent 86% of the state’s population (Adams, Boone, Champaign, Cook, DuPage, Effingham, Fulton, Kane, Kankakee, Kendall, Lake, Lasalle, Livingston, Logan, McDonough, McHenry, McLean, Macoupin, Madison, Peoria, Perry, Rock Island, St. Clair, Sangamon, Tazewell, Vermillion, Will, and Winnebago). Data for violent deaths that occurred in Pennsylvania include 39 counties that represent 82.2% of the state’s population (Adams, Allegheny, Armstrong, Beaver, Berks, Blair, Bradford, Bucks, Cambria, Carbon, Centre, Chester, Clarion, Clearfield, Clinton, Columbia, Crawford, Dauphin, Delaware, Fayette, Forest, Greene, Indiana, Jefferson, Lackawanna, Lancaster, Lehigh, Luzerne, Monroe, Montgomery, Montour, Northampton, Philadelphia, Schuylkill, Union, Wayne, Westmoreland, Wyoming, and York). Data for violent deaths that occurred in California include 21 counties that represent 54% of the state’s population (Amador, Butte, Fresno, Humboldt, Imperial, Kern, Kings, Lake, Los Angeles, Marin, Mono, Placer, Sacramento, San Benito, San Diego, San Francisco, San Mateo, Shasta, Siskiyou, Ventura, and Yolo). Denominators for the rates for these three states (California, Illinois, and Pennsylvania) represent only the populations of the counties from which the data were collected.

** Denominator includes those decedents involved in an incident that was precipitated by another crime.

^††^ Circumstances were unknown for 30 decedents (25 males and five females); total number of legal intervention deaths = 764 (727 males and 37 females).

#### Legal Intervention Deaths and Law Enforcement Officers

For 2018, a total of 39 states and the District of Columbia collected data on 547 law enforcement officers who inflicted fatal injuries in legal intervention deaths from 383 legal intervention incidents. Almost all officers inflicting fatal injuries with known sex (98.7%) were male (96.3%) (Table 9). Age was unknown for 66.2% of officers. Those aged 25–44 years comprised the largest proportion of officers (26.5%), followed by those aged 45–64 (6.2%). Race and ethnicity were unknown for more than one half (56.1%) of officers who inflicted fatal injuries in legal intervention deaths. When race and ethnicity were known, the largest proportion of officers was non-Hispanic White (37.8%), followed by Hispanic (3.1%) and non-Hispanic Black (2.9%).

**TABLE 9:** Number and percentage* of law enforcement officers involved in legal intervention deaths, by age group and race and ethnicity — National Violent Death Reporting System, 39 states and the District of Columbia, 2018^†^

Characteristic	No. (%)
**Age group (yrs)**	
18–24	6 (1.1)
25–44	145 (26.5)
45–64	34 (6.2)
≥65	0 (0)
Unknown	362 (66.2)
**Race/Ethnicity**	
White, non-Hispanic	207 (37.8)
Black, non-Hispanic	16 (2.9)
American Indian or Alaska Native, non-Hispanic	0 (0)
Asian or Pacific Islander, non-Hispanic	0 (0)
Hispanic^§^	17 (3.1)
Unknown	307 (56.1)
**Total**	**547 (100.0)**

* Percentages might not total 100% due to rounding. There were 757 legal intervention incidents. Percentage is based on the number of law enforcement officers (n = 547; male, n= 527; female, n = 13; sex unknown, n = 7) from legal intervention incidents with any information about the officer involved (n = 383; 50.6%). Of officers with known sex, 96.3% were male. Some incidents had more than one suspect.

^†^ Data for all violent deaths were collected in 36 states (Alabama, Alaska, Arizona, California, Colorado, Connecticut, Delaware, Georgia, Illinois, Indiana, Iowa, Kansas, Kentucky, Louisiana, Maine, Maryland, Massachusetts, Michigan, Minnesota, Missouri, Nebraska, Nevada, New Hampshire, New Jersey, New Mexico, New York, North Carolina, Ohio, Oklahoma, Oregon, Pennsylvania, Rhode Island, South Carolina, Utah, Vermont, Virginia, Washington, West Virginia, and Wisconsin), and the District of Columbia. Three states (California, Illinois, and Pennsylvania) collected data from a subset of counties in their state. Data for violent deaths that occurred in Illinois include 28 counties that represent 86% of the state’s population (Adams, Boone, Champaign, Cook, DuPage, Effingham, Fulton, Kane, Kankakee, Kendall, Lake, Lasalle, Livingston, Logan, McDonough, McHenry, McLean, Macoupin, Madison, Peoria, Perry, Rock Island, St. Clair, Sangamon, Tazewell, Vermillion, Will, and Winnebago). Data for violent deaths that occurred in Pennsylvania include 39 counties that represent 82.2% of the state’s population (Adams, Allegheny, Armstrong, Beaver, Berks, Blair, Bradford, Bucks, Cambria, Carbon, Centre, Chester, Clarion, Clearfield, Clinton, Columbia, Crawford, Dauphin, Delaware, Fayette, Forest, Greene, Indiana, Jefferson, Lackawanna, Lancaster, Lehigh, Luzerne, Monroe, Montgomery, Montour, Northampton, Philadelphia, Schuylkill, Union, Wayne, Westmoreland, Wyoming, and York). Data for violent deaths that occurred in California include 21 counties that represent 54% of the state’s population (Amador, Butte, Fresno, Humboldt, Imperial, Kern, Kings, Lake, Los Angeles, Marin, Mono, Placer, Sacramento, San Benito, San Diego, San Francisco, San Mateo, Shasta, Siskiyou, Ventura, and Yolo). Denominators for the rates for these three states (California, Illinois, and Pennsylvania) represent only the populations of the counties from which the data were collected.

^§^ Includes persons of any race.

### Unintentional Firearm Deaths

#### Sex, Age Group, and Race and Ethnicity

For 2018, a total of 39 states and the District of Columbia collected data on 335 incidents involving 337 unintentional firearm deaths. Approximately one half (160; 47.5%; data not shown) of these deaths were self-inflicted, and 129 deaths (38.3%; data not shown) were known to be inflicted by another person; for the remaining 48 deaths (14.2%; data not shown), who inflicted the injury was unknown. Males accounted for 88.1% of decedents (Table 10). Persons aged ≤24 years accounted for approximately one half (54.5%) of all unintentional firearm deaths. Approximately 19.8% of decedents were aged <15 years. The majority of decedents were non-Hispanic White (57.0%) persons, followed by non-Hispanic Black persons (32.3%).

**TABLE 10:** Number and percentage* of unintentional firearm deaths, by selected demographic characteristic of decedent, location of injury, and type of firearm — National Violent Death Reporting System, 39 states and the District of Columbia, 2018^†^

Characteristic	No. (%)
**Sex**	
Male	297 (88.1)
Female	40 (11.9)
**Race/Ethnicity**	
White, non-Hispanic	192 (57.0)
Black, non-Hispanic	109 (32.3)
American Indian or Alaska Native, non-Hispanic	7 (2.1)
Asian or Pacific Islander, non-Hispanic	3 (<1.0)
Hispanic^§^	26 (7.7)
**Age group (yrs)**	
<1	0 (0)
1–4	24 (7.1)
5–9	16 (4.7)
10–14	27 (8.0)
15–19	68 (20.2)
20–24	49 (14.5)
25–29	24 (7.1)
30–34	22 (6.5)
35–44	22 (6.5)
45–54	21 (6.2)
55–64	29 (8.6)
65–74	21 (6.2)
75–84	12 (3.6)
≥85	2 (<1.0)
**Location**	
House or apartment	256 (76.0)
Natural area	23 (6.8)
Motor vehicle	17 (5.0)
Street or highway	7 (2.1)
Hotel or motel	6 (1.8)
Commercial or retail area	3 (<1.0)
Parking lot, public garage, or public transport	3 (<1.0)
Other location^¶^	11 (3.3)
Unknown	11 (3.3)
**Firearm type**	
Handgun	208 (61.7)
Rifle	44 (13.1)
Shotgun	30 (8.9)
Other firearm	1 (<1.0)
Unknown	54 (16.0)
**Total**	**337 (100.0)**

* Percentages might not total 100% due to rounding.

^†^ Data for all violent deaths were collected in 36 states (Alabama, Alaska, Arizona, California, Colorado, Connecticut, Delaware, Georgia, Illinois, Indiana, Iowa, Kansas, Kentucky, Louisiana, Maine, Maryland, Massachusetts, Michigan, Minnesota, Missouri, Nebraska, Nevada, New Hampshire, New Jersey, New Mexico, New York, North Carolina, Ohio, Oklahoma, Oregon, Pennsylvania, Rhode Island, South Carolina, Utah, Vermont, Virginia, Washington, West Virginia, and Wisconsin), and the District of Columbia. Three states (California, Illinois, and Pennsylvania) collected data from a subset of counties in their state. Data for violent deaths that occurred in Illinois include 28 counties that represent 86% of the state’s population (Adams, Boone, Champaign, Cook, DuPage, Effingham, Fulton, Kane, Kankakee, Kendall, Lake, Lasalle, Livingston, Logan, McDonough, McHenry, McLean, Macoupin, Madison, Peoria, Perry, Rock Island, St. Clair, Sangamon, Tazewell, Vermillion, Will, and Winnebago). Data for violent deaths that occurred in Pennsylvania include 39 counties that represent 82.2% of the state’s population (Adams, Allegheny, Armstrong, Beaver, Berks, Blair, Bradford, Bucks, Cambria, Carbon, Centre, Chester, Clarion, Clearfield, Clinton, Columbia, Crawford, Dauphin, Delaware, Fayette, Forest, Greene, Indiana, Jefferson, Lackawanna, Lancaster, Lehigh, Luzerne, Monroe, Montgomery, Montour, Northampton, Philadelphia, Schuylkill, Union, Wayne, Westmoreland, Wyoming, and York). Data for violent deaths that occurred in California include 21 counties that represent 54% of the state’s population (Amador, Butte, Fresno, Humboldt, Imperial, Kern, Kings, Lake, Los Angeles, Marin, Mono, Placer, Sacramento, San Benito, San Diego, San Francisco, San Mateo, Shasta, Siskiyou, Ventura, and Yolo). Denominators for the rates for these three states (California, Illinois, and Pennsylvania) represent only the populations of the counties from which the data were collected.

^§^ Includes persons of any race.

^¶^ Other location includes (in descending order) bar or nightclub; office building; park, playground, or sports or athletic area; farm; and other unspecified location.

#### Location of Injury and Firearm Type

Among unintentional firearm deaths, 76.0% occurred in a house or apartment, followed by a natural area (6.8%) or a motor vehicle (5.0%) (Table 10). The majority of unintentional firearm deaths involved a handgun (61.7%), followed by a rifle (13.1%) or a shotgun (8.9%). In 16.0% of unintentional firearm deaths, the firearm type was unknown.

#### Context and Circumstances of Injury

The context and circumstances of injury were identified in 88.4% of unintentional firearm deaths (Table 10). Among those with context and circumstance information, the most common context of injury was playing with a gun (41.6%) followed by showing the gun to others (15.4%), cleaning the gun (8.7%), and hunting (7.0%) (Table 11). Regarding the circumstances of injury, approximately one fourth (22.1%) of unintentional firearm deaths were caused by a person unintentionally pulling the trigger, 11.7% mistakenly thinking the gun was unloaded, and 6.7% mistakenly thinking the magazine was disengaged.

**TABLE 11:** Number and percentage* of unintentional firearm deaths, by context and circumstance of injury — National Violent Death Reporting System, 39 states and the District of Columbia, 2018^†^

Characteristic	No. (%)
**Context of injury**	
Playing with gun	124 (41.6)
Showing gun to others	46 (15.4)
Cleaning gun	26 (8.7)
Hunting	21 (7.0)
Loading or unloading gun	13 (4.4)
Target shooting	8 (2.7)
Celebratory firing	1 (<1.0)
Other context of injury	72 (24.2)
**Circumstance of injury**	
Unintentionally pulled trigger	66 (22.1)
Thought gun was unloaded	35 (11.7)
Thought unloaded, magazine disengaged	20 (6.7)
Gun was dropped	18 (6.0)
Gun was mistaken for a toy	9 (3.0)
Thought gun safety was engaged	8 (2.7)
Gun fired due to defect or malfunction	6 (2.0)
Bullet ricocheted	3 (1.0)
Gun fired while handling safety lock	2 (<1.0)
Other mechanism of injury	52 (17.4)
**Total^§^**	**298 (88.4)**

* Percentages might exceed 100% because one or more circumstances could have been known per death. Number and percentage are reported when the number of deaths is fewer than five because no particular circumstance identifies a single death. Denominator includes those deaths with one or more precipitating circumstances.

^†^ Data for all violent deaths were collected in 36 states (Alabama, Alaska, Arizona, California, Colorado, Connecticut, Delaware, Georgia, Illinois, Indiana, Iowa, Kansas, Kentucky, Louisiana, Maine, Maryland, Massachusetts, Michigan, Minnesota, Missouri, Nebraska, Nevada, New Hampshire, New Jersey, New Mexico, New York, North Carolina, Ohio, Oklahoma, Oregon, Pennsylvania, Rhode Island, South Carolina, Utah, Vermont, Virginia, Washington, West Virginia, and Wisconsin), and the District of Columbia. Three states (California, Illinois, and Pennsylvania) collected data from a subset of counties in their state. Data for violent deaths that occurred in Illinois include 28 counties that represent 86% of the state’s population (Adams, Boone, Champaign, Cook, DuPage, Effingham, Fulton, Kane, Kankakee, Kendall, Lake, Lasalle, Livingston, Logan, McDonough, McHenry, McLean, Macoupin, Madison, Peoria, Perry, Rock Island, St. Clair, Sangamon, Tazewell, Vermillion, Will, and Winnebago). Data for violent deaths that occurred in Pennsylvania include 39 counties that represent 82.2% of the state’s population (Adams, Allegheny, Armstrong, Beaver, Berks, Blair, Bradford, Bucks, Cambria, Carbon, Centre, Chester, Clarion, Clearfield, Clinton, Columbia, Crawford, Dauphin, Delaware, Fayette, Forest, Greene, Indiana, Jefferson, Lackawanna, Lancaster, Lehigh, Luzerne, Monroe, Montgomery, Montour, Northampton, Philadelphia, Schuylkill, Union, Wayne, Westmoreland, Wyoming, and York). Data for violent deaths that occurred in California include 21 counties that represent 54% of the state’s population (Amador, Butte, Fresno, Humboldt, Imperial, Kern, Kings, Lake, Los Angeles, Marin, Mono, Placer, Sacramento, San Benito, San Diego, San Francisco, San Mateo, Shasta, Siskiyou, Ventura, and Yolo). Denominators for the rates for these three states (California, Illinois, and Pennsylvania) represent only the populations of the counties from which the data were collected.

^§^ Circumstances were unknown for 39 decedents; total number of unintentional firearm decedents = 337.

### Deaths of Undetermined Intent

#### Sex, Age Group, and Race and Ethnicity

For 2018, a total of 39 states and the District of Columbia collected data on 4,869 incidents involving 4,902 deaths of undetermined intent (Supplementary Table S1, https://stacks.cdc.gov/view/cdc/112767). The overall rate of deaths of undetermined intent was 2.1 per 100,000 population. The rate of deaths of undetermined intent was higher among males (2.8 per 100,000 population) than among females (1.4 per 100,000 population) (Supplementary Table S4, https://stacks.cdc.gov/view/cdc/112767). More than one half (59.3%) of deaths of undetermined intent were among adults aged 35–64 years. The rate of deaths of undetermined intent was highest among adults aged 45–54 years (3.6 per 100,000 population), followed by adults aged 30–34 years (3.5 per 100,000 population), 35–44 years (3.2 per 100,000 population), and 55–64 years (2.9 per 100,000 population). Although non-Hispanic White persons accounted for the majority (67.9%, 2.2 per 100,000 population) of deaths of undetermined intent, non-Hispanic Black persons had the highest rate (3.5 per 100,000 population). Among males, non-Hispanic Black persons (5.6 per 100,000 population) and non-Hispanic AI/AN persons (3.8 per 100,000 population) had the highest rate of deaths of undetermined intent. Among females, non-Hispanic Black persons had the highest rate of deaths of undetermined intent (1.7 per 100,000 population), followed by non-Hispanic White persons (1.6 per 100,000 population).

#### Method and Location of Injury

Poisoning was the most common method of injury in deaths of undetermined intent (68.3%) (Supplementary Table S4, https://stacks.cdc.gov/view/cdc/112767). No other known method accounted for >4.6% of deaths The majority of deaths of undetermined intent occurred in a house or apartment (66.1%), followed by a natural area (5.0%) or a street or highway (3.8%).

#### Toxicology Results of Decedent

Tests for antidepressants, benzodiazepines, and opioids were conducted for 35.9%, 40.8%, and 73.8% of decedents, respectively (Supplementary Table S5, https://stacks.cdc.gov/view/cdc/112767). Results for antidepressants were positive in 56.4% of decedents and benzodiazepines were positive in 46.7% of decedents tested for those substances. Results for opioids (including illicit or prescription) were positive in 77.8% of decedents tested.

#### Precipitating Circumstances

Circumstances were identified in 80.5% of deaths of undetermined intent (Supplementary Table S6, https://stacks.cdc.gov/view/cdc/112767). Among deaths of undetermined intent with known circumstances, 35.9% of decedents had a current diagnosed mental health problem; depression or dysthymia (55.1%), anxiety disorder (25.7%), and bipolar disorder (22.3%) were the most common diagnoses. Among deaths of undetermined intent, 22.6% were receiving mental health treatment at the time of death and 7.7% of decedents had a current depressed mood. Substance use problems (other than alcohol) (69.0%) and alcohol problems (25.2%) were the most commonly reported circumstances. Physical health problems (11.7%) and a recent or impending crisis during the preceding or upcoming 2 weeks (9.5%) were other life stressors identified in deaths of undetermined intent. Among decedents, 9.7% had a history of suicidal thoughts or plans, 6.9% had a history of suicide attempt, and 4.1% had disclosed intent to die by suicide.

### Violent Deaths in Puerto Rico

For 2018, Puerto Rico collected data on 880 incidents involving 975 deaths. Homicide (672; 68.9%) accounted for the highest rate of violent death (21.0 per 100,000 population), followed by suicide (266; 27.3%; 9.2 per 100,000 population aged ≥10 years) (Supplementary Table S15, https://stacks.cdc.gov/view/cdc/112767).

#### Homicides

##### Sex, Age Group, and Race and Ethnicity

For 2018, a total of 619 homicides among males and 53 homicides among females were reported in Puerto Rico (Supplementary Table S15, https://stacks.cdc.gov/view/cdc/112767). The overall homicide rate for males was 12.8 times the rate for females (40.8 versus 3.2 per 100,000 population). Among males, the homicide rate was 105.0 per 100,000 population among those aged 18–29 years and 81.1 per 100,000 population among those aged 30–44 years. Most (95.1%) homicide victims were Hispanic.

##### Method, Location of Injury, and Victim-Suspect Relationship

A firearm was used in most (90.3%) of homicides (Supplementary Table S15, https://stacks.cdc.gov/view/cdc/112767). A firearm was the most common method used in homicides of both males (91.6%) and females (75.5%); however, the firearm homicide rate for males (37.4 per 100,000 population) was 15.6 times the rate for females (2.4 per 100,000 population). Among males, a street or highway was the most common location (49.1%) of homicides, whereas a house or apartment was the most common location (43.4%) of homicides for females.

The victim-suspect relationship was known for 33.6% of homicides (Supplementary Table S15, https://stacks.cdc.gov/view/cdc/112767). When the relationship was known, the suspect for male victims was most often another person known to the victim (46.8%) but the exact nature of the relationship was unclear, whereas the suspect for approximately two thirds (63.9%) of female victims was a current or former intimate partner.

##### Toxicology Results of Decedent

Tests for alcohol were conducted for 99.3% of homicide decedents (Supplementary Table S16, https://stacks.cdc.gov/view/cdc/112767). Among those with positive results for alcohol (36.0%), 52.1% had a BAC ≥0.08 g/dL. Tests for cocaine, marijuana, and opioids were conducted for 99.1%, 67.6%, and 99.0% of decedents, respectively. Results for cocaine, marijuana, and opioids were positive in 14.6%, 36.3%, and 3.3% of decedents tested, respectively.

##### Precipitating Circumstances

Precipitating circumstances were identified in 90.6% of homicides (Supplementary Table S17, https://stacks.cdc.gov/view/cdc/112767). Among males, 52.4% of homicides involved illicit drugs and 51.7% were gang related. More than one fourth (27.0%) of homicides among males involved drive-by shootings. Intimate partner violence was identified as a contributing factor in 11.0% of homicides overall; a larger proportion of homicides among females (46.0%) was precipitated by intimate partner violence than among males (7.9%).

#### Suicides

##### Sex, Age Group, and Race and Ethnicity

For 2018, a total of 266 suicides among persons aged ≥10 years (231 suicides among males and 35 suicides among females) were reported in Puerto Rico (Supplementary Table S18, https://stacks.cdc.gov/view/cdc/112767). The suicide rate for males was 7.3 times the rate for females (16.9 versus 2.3 per 100,000 population aged ≥10 years). The suicide rate was 25.8 per 100,000 population among males aged 30–44 years and 21.8 per 100,000 population among males aged 45–64 years. Most (93.2%) suicide decedents were Hispanic.

##### Method and Location of Injury

Hanging, strangulation, or suffocation was the most commonly used method for suicide among both males (67.5%) and females (68.6%) (Supplementary Table S18, https://stacks.cdc.gov/view/cdc/112767). A firearm was used in 19.0% of suicides among males. The most common location where a suicide took place was a house or apartment for both males (78.4%) and females (82.9%).

##### Toxicology Results of Decedent

Tests for alcohol were conducted for 98.9% of suicide decedents (Supplementary Table S19, https://stacks.cdc.gov/view/cdc/112767). Among those with positive results for alcohol (30.4%), 62.5% had a BAC ≥0.08 g/dL. Tests for cocaine, marijuana, and opioids were conducted for 98.9%, 62.0%, and 98.5% of decedents, respectively. Results for cocaine were positive in 9.9% of decedents tested, and results for marijuana were positive in 8.5% of decedents tested.

##### Precipitating Circumstances

Circumstances were identified in 93.2% of suicides (Supplementary Table S20, https://stacks.cdc.gov/view/cdc/112767). Overall, a mental health problem was the most common circumstance among suicide decedents, with 51.2% experiencing a depressed mood at the time of death and 42.3% having a current diagnosed mental health problem.

Among males, 51.2% of suicide decedents had a current depressed mood, and 38.1% had a current diagnosed mental health problem. Depression or dysthymia was most often the mental health diagnosis experienced by male suicide decedents with a diagnosed mental health problem (80.5%), followed by schizophrenia (15.9%). One fourth (25.6%) of male suicide decedents had a history of ever being treated for a mental health problem. More than one third (34.0%) of male suicide decedents had a history of expressing suicidal thoughts and plans, and 26.5% had a history of attempting suicide. Other precipitating circumstances for male suicides included intimate partner problems (21.9%), physical health problem (20.5%), an argument or conflict (9.8%), and a crisis during the previous or upcoming 2 weeks (8.8%).

Among female suicide decedents, 51.5% had current depressed mood, and 69.7% had a current diagnosed mental health problem. Depression or dysthymia was most often the mental health diagnosis experienced by female suicide decedents who had a diagnosed mental health problem (69.6%). Among female decedents, 42.4% had a history of ever being treated for a mental health problem, and 48.5% had a history of attempting suicide.

## Discussion

Violent deaths affect males and females and persons of all ages, races, and ethnicities. NVDRS data on specific manners of violent death can be used to describe characteristics of and inequities experienced by populations particularly affected by fatal violence. NVDRS data can also be used to identify cross-cutting risk factors for multiple forms of violence. These details increase the knowledge base about the circumstances associated with violence and can assist public health authorities and their partners in developing and guiding effective, data-driven approaches to violence prevention.

The occurrence of violent death varies greatly across states, the District of Columbia, and Puerto Rico (*1*). This report summarizes data on violent deaths that occurred in 2018 in 39 states and the District of Columbia, representing 72.0% of the U.S. population and accounting for 72.9% of violent deaths in the United States in 2018 (*1*), and Puerto Rico. In 2019, NVDRS expanded data collection to include all 50 states, the District of Columbia, and Puerto Rico, providing more comprehensive, accessible, and actionable violent death information that can be used to guide the development of evidence-based violence prevention efforts at local, regional, state, and national levels. Expanding NVDRS to a nationwide system also contributes to the national prevention initiative *Healthy People 2020* objectives to increase the number of states that link data on violent deaths from death certificates, coroner or medical examiner reports, and law enforcement reports at state and local levels and the *Healthy People 2030* objectives to reduce the number of suicides, homicides, and firearm-related deaths (*12*,*13*).

Violence is preventable, and reducing violent deaths in communities is possible with evidence-based approaches, such as those outlined in CDC’s technical packages for violence prevention (*14*). CDC developed technical packages to assist communities in identifying violence prevention approaches that are based on the best available evidence. The five technical packages describe strategies, approaches, and specific programs, practices, and policies with evidence to reduce the risk for suicide, youth violence, child abuse and neglect, intimate partner violence, and sexual violence. Each technical package considers the multifaceted and interactive effects of different levels of the social ecology, including individual, relationship, family, school, and community factors that influence violence-related outcomes. NVDRS gathers ongoing, systematic, and consistent data on violent deaths that can be used by violence prevention experts within their communities to guide planning and implementation and track outcomes of violence prevention strategies and approaches.

### Suicides

#### Suicide Circumstances

Suicide rates were highest among males and adults aged 45–64 years. Approximately one third of suicide decedents had a history of suicidal thoughts or plans, and approximately one fourth had disclosed their suicidal intent. Mental health problems were the most commonly identified circumstance; however, approximately one half of suicide decedents did not have a known mental health condition at the time of their death. Past suicidal behavior and mental health problems are important risk factors for suicide (*15*), and these circumstances are well documented as important risk factors to target in suicide prevention (*15*,*16*). Approximately one third of suicide decedents were known to be receiving treatment at the time of death, pointing to a gap between those receiving treatment and those who would likely benefit from it. Multiple factors contribute to the risk for suicide (*17*), and the findings in this report indicate that intimate partner problems, recent or impending crises, and physical health problems also were common precipitating circumstances. A high prevalence of alcohol use was observed among suicide decedents tested for substances, especially those with BAC ≥0.08g/dL. Alcohol use is a robust predictor of suicidal behavior (*18*), victimization (*19*), and interpersonal violence perpetration (*20*,*21*). Intoxication can lead to disinhibition, enhance feelings of hopelessness and depression, and impair judgment, which can lead to impulsive behaviors (*16*).

Another factor that might contribute to the risk for suicide is access to lethal means (*15*). Similar to rates observed for alcohol and antidepressants, toxicology results for opioids (illicit or prescription) were positive in approximately one fourth of suicide decedents who were tested for these substances. Opioid overdose has been recognized as an epidemic (*22*). CDC published the *Guideline for Prescribing Opioids for Chronic Pain* to provide recommendations for primary care physicians who are prescribing opioids for chronic pain outside of active cancer treatment, palliative care and end-of-life care to improve communication between clinicians and patients about the risks and benefits of opioid therapy for chronic pain, support safer prescribing practices, and reduce the risks associated with long-term opioid therapy including opioid use disorder, overdose, and death (*23*). Previous research suggests that chronic pain might be a contributor to suicide; for this reason, these guidelines also aim to ensure that patients receive appropriate care for pain (*24*). Building on previous CDC programs focused on opioid overdose and injury prevention, CDC also has implemented comprehensive surveillance and prevention activities through *Overdose Data to Action* to support state and local health departments in collecting and reporting more timely and complete data on overdose morbidity and mortality and using the data to inform prevention and response efforts (*25*–*27*). Other activities to address the opioid overdose epidemic include expanding naloxone availability and access to treatment with medications for opioid use disorder, enhancing public health and public safety partnerships, and maximizing the ability of health systems to link persons to treatment and harm-reduction services (*25*–*28*). A firearm was the most common method used in suicides. Lethal means, such as firearms, provide limited opportunity for intervention and have high case-fatality rates (*15*). Creating protective environments by reducing access to lethal means among persons at-risk can be an effective strategy to prevent suicide (*15*).

#### Racial and Ethnic Inequities in Suicide Rates

Demographic variations persist in the manner of death from violence-related injuries. Suicides comprise the majority of violent deaths collected in NVDRS and occur at higher rates among non-Hispanic AI/AN and White persons. The findings regarding suicide rates experienced by non-Hispanic AI/AN persons warrant attention to the contextual factors that might contribute to higher rates of suicide, such as barriers to accessing mental health care, exposure to the suicide of a friend or family member as a contributing factor to one’s own death by suicide, and alcohol and substance use (*29*). AI/AN persons’ experiences with historical trauma related to the intergenerational, collective, and cumulative impact of colonialism and ongoing inequities including discrimination, disparaging stereotypes, and microaggressions can contribute to risk for suicide (*30*,*31*). Challenges related to suicide, alcohol, and substance use are not inherent to AI/AN culture but should be interpreted within the context of historical racism and ongoing inequities. The heterogeneity among persons and groups that identify as AI/AN also should be acknowledged (*29*,*30*).

#### Suicide Prevention Strategies

States participating in NVDRS have used their data to support prevention efforts. NVDRS programs often partner with collaborators to provide data on violent deaths, and those collaborators in turn use the data to guide prevention efforts. For example, Arizona and Wisconsin use their VDRS data to support suicide prevention efforts within their respective states. Arizona VDRS partners with Arizona’s statewide *Be Connected *initiative to provide customized community-level data on veteran suicide deaths in Arizona. *Be Connected* directly engages in veteran suicide prevention, including staffing a support line, providing resource matching and navigation, and training for the community (*32*). This partnership demonstrates the applied use of NVDRS data for Arizona, and consequently allows for proactive outreach to and engagement with veterans at risk for suicide. 

Wisconsin VDRS used multiple years of data (2013–2017) to identify important risk and protective factors and subsequently develop a comprehensive suicide prevention plan for Wisconsin. Wisconsin VDRS found that during 2013–2017, suicide rates were highest among non-Hispanic White and AI/AN persons (*33*). Non-Hispanic groups had higher rates of suicide than the Hispanic population, and nonfatal self-harm injury rates were highest among non-Hispanic Black and AI/AN persons (*33*). Rural counties had higher suicide rates compared with urban and suburban counties (*33*). In addition, 25% of suicide decedents had a previous suicide attempt, and 20% had a reported job problem, financial problem, or both (*33*). These findings demonstrated the impact of suicide in Wisconsin and prompted the development of the Wisconsin Suicide Prevention Plan (*33*), which is a comprehensive plan designed to increase the effectiveness of suicide prevention efforts in Wisconsin. In addition, to address high rates of suicide among at-risk populations, the plan proposes to expand access to services for mental health and substance use treatment (including physical health care), support innovative ways to expand access to care, including technologies and peer-led or other nonclinical support services, and increase the public’s knowledge of suicides, which includes recognizing warning signs and preparedness to support and respond to persons at risk. This plan highlights the importance of using the best available data; mobilizing coordinated, effective efforts by state agencies, local suicide prevention coalitions, and other partners; facilitating statewide impact of evidence-based and best practices; and monitoring the progress of the prevention plan at the state level, all of which might help to reduce the overall number of suicides in Wisconsin.

CDC’s suicide prevention technical package describes seven strategies for reducing suicide and suicidal behaviors: 1) strengthen economic supports, 2) strengthen access to and delivery of suicide care, 3) create protective environments, 4) promote connectedness, 5) teach coping and problem-solving skills, 6) identify and support persons at risk, and 7) decrease harms and prevent future risk (*14*). These strategies support the goals and objectives of the National Strategy for Suicide Prevention (NSSP), which is a comprehensive national agenda for suicide prevention (*34*), and the National Action Alliance for Suicide Prevention’s priority to strengthen community-based prevention (*35*). NVDRS is relevant to NSSP’s goals of increasing timeliness and usefulness of surveillance systems related to suicide prevention and evaluating outcomes and effectiveness of suicide prevention interventions. The suicide prevention technical package includes examples of specific approaches that communities can implement to advance each strategy. The findings in this report underscore the importance of approaches outlined in the suicide technical package, such as social-emotional learning programs, enhancing parenting skills and family relationships, and treatment for persons at risk for suicide and treatment to prevent reattempts.

### Homicides

#### Homicides of Infants and Children

Infants experienced a high homicide rate, highlighting the need for prioritizing child abuse and neglect prevention and intervention strategies to reduce risk for morbidity and mortality. Child abuse and neglect are often associated with immediate physical injuries, emotional and psychological problems, involvement in risky health behaviors later in life, and a host of broader physical health challenges and long-term health consequences (*36*).

CDC’s child abuse and neglect prevention technical package identified the following evidence-based strategies and approaches: 1) strengthening economic supports for families, 2) changing social norms to support parents and positive parenting, 3) providing quality care and education early in life, 4) enhancing parenting skills to promote healthy child development, and 5) intervening to decrease harms and prevent future risk (*36*). Child abuse and neglect are preventable, and the specific approaches described in the technical package can help create safe, stable, and nurturing relationships and environments (*37*) to prevent homicides of infants and children as well as nonfatal child maltreatment.

#### Racial and Ethnic Inequities in Homicide Rates

Racial and ethnic minority groups experience inequitable rates of violent injury and homicide, particularly among youths and young adult males (*38*). In the United States, homicide rates were highest among non-Hispanic Black and AI/AN persons. In Puerto Rico, where 95.1% of homicide victims were Hispanic, the homicide rate was more than double the suicide rate. Male homicide victims in Puerto Rico were predominantly Hispanic (94.8%) and experienced homicide rates similar to and even exceeding the homicide rates experienced by non-Hispanic Black, non-Hispanic AI/AN, and Hispanic males in the United States. Racial and ethnic inequities in exposure to violence are pervasive and persistent, and the elimination of these inequities should be prioritized (*38*). Racial and ethnic minority groups are disproportionately exposed to systemic inequities such as residential segregation, concentrated disadvantage, stress from experiencing racism, limited access to the best educational and employment opportunities, and other conditions that increase the risk for experiencing violence (*38*–*40*). For example, homicide rates for males in Puerto Rico have been attributed, in part, to persons living in communities that have been marginalized and the socioeconomic incentives of being involved in illegal means of income that are associated with high risks for violence (*41*). Racial and ethnic minority youths often live in communities with concentrated poverty, stressed economies, residential instability, neighborhood disorganization, and low community cohesion and informal controls (*35*,*36*). These conditions are associated with violence and violence-related injuries (*39*). By addressing the structural, societal, and community-level contexts that serve as risk factors for violence, prevention efforts can have broad and sustained effects (*3*,*41*,*42*).

NVDRS programs have used their local data to examine violence-related disparities in their states. For example, the Illinois VDRS used 2016–2017 Illinois VDRS data to examine the intersection of homicide and poverty at the county-level and found a relation between homicide and poverty in two thirds of the counties that were examined (*43*). Similarly, Illinois VDRS examined firearm-related homicide rates among non-Hispanic Black males aged 15–19 years in Chicago during 2013–2017 and found that the 2017 firearm-related homicide rate among Black males in Chicago aged 15–19 years was 74% higher than that in 2013 (*44*). During 2013–2017, non-Hispanic Black males in Chicago aged 15–19 years had an increased risk for firearm-related homicide compared with all persons in the United States and Chicago in this age group. Although firearm-related homicide risk for non-Hispanic Black males aged 15–19 years fluctuated during 2013–2017, in 2017, non-Hispanic Black males aged 15–19 years in Chicago were 13.7 times more likely to be victims of firearm-related homicide compared with non-Black males in this age group (*44*). These findings indicate increasing disparities between young non-Hispanic Black males in Chicago and all other young males in the United States. In conjunction with other data sources, NVDRS data can help states identify and target salient neighborhood- and community-level factors related to violence, which can contribute to greater population-level decreases in violence through the reduction and elimination of systemic inequities (*45*). CDC’s youth violence prevention technical package outlines several community- and societal-level programs and approaches (*14*), such as Baltimore’s Safe Streets (*46*), Crime Prevention Through Environmental Design (*47*), business improvement districts (*48*,*49*), and policies such as the Earned Income Tax Credit (EITC) (*50*,*51*). For example, enhancing household financial security through tax credits, such as the EITC, can help families raise their income while incentivizing work or counterbalancing the costs of child-rearing and help create home environments that encourage healthy development (*50*,*51*). Evaluations of these programs and policies have confirmed the value of using these types of approaches to reduce the risk for violence and promote protective community environments (*14*). Evidence also suggests that these approaches and other universal policies that focus on general community improvements can have a substantial impact on decreasing racial and ethnic inequities in violence (*39*).

#### Intimate Partner Violence–Related Homicide

Homicides among males were most often preceded by an argument or conflict or precipitated by another crime, and the suspect was most often an acquaintance or friend. In contrast, approximately 45% of homicides among females were related to intimate partner violence, and a current or former intimate partner was identified as the suspect for approximately one half of female homicide victims with known suspects. These findings were consistent with another NVDRS report that highlighted the differential impact of intimate partner violence–related homicides among young and racial and ethnic minority women (*52*). Intimate partner violence affects millions of persons in the United States each year (*53*). Estimates from the 2015 National Intimate Partner and Sexual Violence Survey indicated that approximately 80 million persons in the United States have experienced intimate partner violence (e.g., contact sexual violence, physical violence, and stalking by an intimate partner) at some point in their lives, and approximately 12 million persons experienced intimate partner violence in the previous 12 months (*53*).

Although approximately one half (49.2%) of homicides involving intimate partner violence were perpetrated by suspects aged 25–44 years, among suspects aged 45–64 years and those aged ≥65 years, the victim was most often a current or former intimate partner. For example, suspects aged ≥65 years accounted for only 8.5% of suspects involved in intimate partner violence–related homicides overall; however, 58.2% of suspects within this age group were involved in intimate partner violence–related homicides. Studies examining older adult perpetration of intimate partner homicide have highlighted the potential influence of factors such as relationship discord, impending separation, caregiver-related stressors, and physical and mental health problems have on intimate partner violence (*54*,*55*). Intimate partner violence–related homicides warrant further research to determine the contextual factors and characteristics of these fatal incidents and how these contextual factors might vary by age group.

NVDRS programs have used their data to examine intimate partner violence–related deaths to support prevention efforts (*56*). Data from the South Carolina VDRS were used to examine intimate partner homicides that occurred in South Carolina during 2017 (*56*). South Carolina VDRS found that 12% of all homicides that occurred in 2017 were intimate partner violence related, with females accounting for 52% of intimate partner violence–related homicide victims. In addition, approximately one in three intimate partner violence–related homicides occurred during an argument, and an argument contributed to the death of 44% of males and 41% of female homicide victims in South Carolina in 2017. These data were shared with domestic violence prevention collaborators in South Carolina to bolster their efforts in reducing intimate partner violence–related deaths.

CDC’s intimate partner violence prevention technical package outlines several strategies for programs and policies to prevent intimate partner violence and to decrease harms (*57*). Strategies and approaches to prevent and reduce intimate partner violence might occur across different levels of the social ecology, such as engaging men and boys as allies (*57*,*58*); disrupting developmental pathways toward intimate partner violence; creating protective school, workplace, and neighborhood environments (*57*); teaching youths about safe and healthy relationships (*57*,*58*); empowering bystanders; and strengthening economic supports to families (*57*). Prevention efforts can help change harmful gender norms that condone violence and the societal conditions that serve to maintain those norms (*57*,*59*).

#### Homicide Suspects

Most homicide suspects with known age were males aged 18–44 years, which is consistent with previous studies describing homicide suspect demographics (*60*). Most suspects were accused of fatally injuring an acquaintance or friend, or a current or former intimate partner or spouse. The data provide more insight into the potential context of homicides when victim-suspect relationships are examined by age group. For example, among persons aged ≤24 years, suspects were most often accused of fatally injuring an acquaintance or friend, stranger, or other person whom they knew but who was not a relative. For suspects aged ≥24 years, increasingly larger proportions of current or former intimate partners or spouses were reflected in the victim-suspect relationship than proportions of other types of victim-suspect relationships. Attempting suicide after a homicide was another prominent contextual factor among those aged ≥45 years. The considerable prevalence of current or former intimate partners as victims and attempted suicide after the homicide are consistent with a previous study that identified having a history of intimate partner conflict as common among homicide-suicide incidents (*60*).

A mental health problem was noted as a direct contributing factor to the homicide for <5% of suspects, which challenges public perceptions of associations between mental health problems and violent behavior (*61*). Exploring contexts in which mental health problems are a contributing factor and dispelling stigma that can lead to discrimination and present barriers to life opportunities for those living with persistent mental health challenges are important (*61*). Approximately 12% of suspects were noted as having contact with law enforcement within the 12 months before the homicide. Prior contact with law enforcement included instances in which the police were called, regardless of whether an arrest was made (*6*). This finding warrants future research to determine the extent and nature of prior law enforcement contact and the potential opportunity for law enforcement to disrupt pathways toward escalating forms of violence, including homicide.

Additional research to examine how incident contexts and risk and protective factors for homicide perpetration vary across age groups can help guide approaches to violence prevention and identify potential intervention points for reducing onset or progression of violence within relationships and communities. Individual, relationship, and community-level interventions that prevent initial violence perpetration or escalating violence are an important part of a comprehensive approach to violence prevention as described in CDC’s technical packages for violence prevention.

#### Legal Intervention Deaths

NVDRS collects more complete information than other data sources on legal intervention deaths (*62*). The rate of legal intervention death was highest among non-Hispanic AI/AN persons, and the rate among non-Hispanic Black males was 2.6 times that of their non-Hispanic White male counterparts, a finding consistent with previous studies (*63*,*64*). Racial and ethnic inequities in fatal police shootings have been examined in violence literature (*63*,*65*–*67*) and have been attributed to factors such as increased police contact caused by more traffic stops, a higher presence of law enforcement in racial and ethnic minority communities, and race-based bias and perceptions of threat. More analyses are needed to increase knowledge about the magnitude and circumstances of these deaths and for developing appropriate prevention strategies and monitoring their effectiveness. Other studies have provided a review of strategies for decreasing legal intervention deaths, such as increasing training in conflict de-escalation and tactical disengagement, and training to reduce potential bias in law enforcement officers’ responses to suspects (*63*,*65*). After a number of high-profile legal intervention deaths including the murder of George Floyd in 2020, several policy changes have been proposed to reduce police violence and enhance relationships between law enforcement and the communities they serve. These proposals include strengthening and monitoring local police use-of-force policies, recruiting a diverse police force, and training officers in appropriate and safe interactions with the community (*68*).

A unique strength of the NVDRS surveillance system is the ability to capture data on suspects, including characteristics of law enforcement officers involved in legal intervention deaths (*2*,*63*). Although 49.4% of 2018 NVDRS legal intervention incidents had unknown demographic and circumstance information for the officers involved, the available information provided some insight into the officers involved in violent deaths that occurred in the line of duty. When demographic information was known, law enforcement officers involved in legal intervention deaths were most frequently non-Hispanic White males aged 25–44 years. The demographics of law enforcement officers involved in legal intervention deaths are consistent with demographics of the U.S. police force, which tends to be predominantly comprised of non-Hispanic White males (*69*). Although not examined in the current report, a previous study examining characteristics of officers involved in legal intervention deaths found associations between officer use of lethal force and demographic characteristics such as race, age, sex, and education as well as previous use of force (*70*). Given previous findings on officers involved in legal intervention deaths and the importance of NVDRS for capturing information on legal intervention deaths, researchers have called on NVDRS to increase the completeness of demographic information on officers involved in these deaths (*63*,*70*).

#### Unintentional Firearm Deaths

NVDRS has been recognized as a reliable source of data on unintentional firearm deaths (*71*) and for its ability to provide details about victims and shooters (*72*). Approximately one half of unintentional firearm deaths were self-inflicted; however, approximately one third were known to have been inflicted by another person. Most of these deaths occurred while playing with a gun, accidentally pulling the trigger, or thinking the gun was unloaded, which are of concern, particularly among children (*73*); these findings highlight the importance of safe storage practices and education about safe handling of firearms (*74*).

## Limitations

The findings in this report are subject to at least seven limitations. First, NVDRS data are available from a limited number of states, the District of Columbia, and Puerto Rico and therefore are not nationally representative. In addition, California, Illinois, and Pennsylvania data were from a subset of counties and are not representative of all violent deaths occurring in these states. However, Illinois and Pennsylvania contributed data that represent a very high percentage of the state populations (86.0% and 82.2%, respectively), and all of these states include a mix of data from large urban population centers and smaller, more rural counties.

Second, the availability, completeness, and timeliness of data depend on partnerships among VDRS programs and local health departments, vital statistics registrars’ offices, coroners and medical examiners, and law enforcement personnel. Data sharing and communication among partners are particularly challenging when states and U.S. territories have independent county coroner systems rather than a centralized coroner or medical examiner system, numerous law enforcement jurisdictions, or both. NVDRS incident data might be limited or incomplete for areas in which these data-sharing relations are not fully developed. Partnerships with local vital statistics registrars’ offices usually are more established because they are part of the public health infrastructure. As part of an active surveillance system, VDRS programs work closely with local vital registrars’ offices to identify deaths meeting the NVDRS case definition and to avoid cases being missed or inappropriately included. CDC also monitors case ascertainment and variable completeness through regular technical assistance calls, which include an internal data quality dashboard in the web-based system that is updated in real-time. Overall, core variables that represent demographic characteristics (e.g., age, sex, and race and ethnicity) and manner of death were known for >99.5% of cases.

Third, toxicology data are not collected consistently across all states, the District of Columbia, and Puerto Rico or for all alcohol and drug categories. In addition, toxicology testing is not conducted for all decedents; thus, the percentages of decedents with positive results for specific substances might be affected by testing practices in coroner or medical examiner offices (*75*).

Fourth, abstractors are limited to the data included in the investigative reports they receive. For example, beyond basic demographics, suspect data are often incomplete or unavailable, which might result in an underestimate of suspect circumstances. In addition, reports might not fully reflect all information known about an incident, particularly for homicides and legal intervention deaths, when data are less readily available until a full investigation and adjudication are completed. In the current report, demographic information regarding the race and ethnicity, sex, and age of homicide suspects and officers involved in legal intervention deaths was incomplete for some incidents. How this unknown information is distributed and whether missing data are related to reporting biases is unclear.

Fifth, case definitions present challenges when a single death is classified differently in different documents (e.g., unintentional firearm death in a law enforcement report, homicide in a coroner or medical examiner report, and undetermined on the death certificate). NVDRS abstractors reconcile these discrepancies using standard NVDRS case definitions and select a single manner of death based on all source documents (*6*).

Sixth, variations in coding occur depending on the abstractor’s level of experience. For this reason, CDC provides extensive abstractor guidance and training, a coding manual to promote standardized data collection (*6*), and data validation checks. As part of their internal data quality efforts, VDRS programs are required to reabstract at least 5% of cases to examine consistency in coding and identify training needs of data abstractors. 

Finally, medical and mental health information (e.g., type of condition and whether the decedent was receiving treatment) often are not captured directly from medical records but from coroner or medical examiner reports and the decedent’s family members and friends. Therefore, the completeness and accuracy of this information are limited to the knowledge of the informant.

## Conclusion

Public health surveillance is the foundation for public health practice (*76*). Monitoring the prevalence of violence-related fatal injuries, defining priorities, and guiding violence prevention activities are essential parts of public health surveillance (*66*). In 2018, NVDRS received funding for nationwide expansion. As of 2019, all 50 states, the District of Columbia, and Puerto Rico participate in NVDRS, a move toward achieving the goal of providing nationally representative data. This expansion makes violent death information available for local communities to develop prevention efforts and allow for the system’s capacity to measure the need for and effects of violence prevention policies, programs, and practices at the national level.

## Footnotes

* The name of the Institute of Medicine was changed to the National Academy of Medicine, effective July 1, 2015.

† To be included in NVDRS, deaths of undetermined intent must have some evidence of the possibility that the intent was purposeful, including use of a weapon or other evidence that force was used to inflict the injury. Most commonly, the coroner or medical examiner is unsure whether the death was a suicide or unintentional.

§ California began collecting data in 2005 but ended data collection in 2009. In 2018, 21 California counties (Amador, Butte, Fresno, Humboldt, Imperial, Kern, Kings, Lake, Los Angeles, Marin, Mono, Placer, Sacramento, San Benito, San Mateo, San Diego, San Francisco, Shasta, Siskiyou, Ventura, and Yolo) contributed data to NVDRS.

¶ Frequencies and rates of violent deaths included in this report differ slightly from the frequencies and rates of violent deaths reported by WISQARS, which excludes nonresident deaths that occur in participating states and the District of Columbia (i.e., occurrent deaths). NVDRS tracks both resident and occurrent violent deaths in the overall data set, and the numbers in this report reflect both. VDRS programs are expected to collect information on violent deaths among their residents, wherever they occur, and fatal violent injuries occurring within their borders irrespective of the decedent’s residence status. If the states of residence and injury occurrence are both participating NVDRS states, the state of injury occurrence is responsible for collecting the information. Making this differentiation of responsibility avoids duplicate reporting.

** Drug involvement is defined as drug dealing, drug trade (i.e., the buying, selling, or passing of drugs in exchange for goods or money), or drug use that is suspected to have played a role in precipitating the incident.
